# Transcriptomic Analysis of Murine Embryos Lacking Endogenous Retinoic Acid Signaling

**DOI:** 10.1371/journal.pone.0062274

**Published:** 2013-04-24

**Authors:** Marie Paschaki, Carole Schneider, Muriel Rhinn, Christelle Thibault-Carpentier, Doulaye Dembélé, Karen Niederreither, Pascal Dollé

**Affiliations:** 1 Developmental Biology and Stem Cells Department, Institut de Génétique et de Biologie Moléculaire et Cellulaire (IGBMC), Centre National de la Recherche Scientifique (Unité Mixte de Recherche 7104), Institut National de la Santé et de la Recherche Médicale (Unité 964), Université de Strasbourg, Illkirch-Strasbourg, France; 2 Biochips platform, Institut de Génétique et de Biologie Moléculaire et Cellulaire (IGBMC), Centre National de la Recherche Scientifique (Unité Mixte de Recherche 7104), Institut National de la Santé et de la Recherche Médicale (Unité 964), Université de Strasbourg, Illkirch-Strasbourg, France; Laboratoire de Biologie du Développement de Villefranche-sur-Mer, France

## Abstract

Retinoic acid (RA), an active derivative of the liposoluble vitamin A (retinol), acts as an important signaling molecule during embryonic development, regulating phenomenons as diverse as anterior-posterior axial patterning, forebrain and optic vesicle development, specification of hindbrain rhombomeres, pharyngeal arches and second heart field, somitogenesis, and differentiation of spinal cord neurons. This small molecule directly triggers gene activation by binding to nuclear receptors (RARs), switching them from potential repressors to transcriptional activators. The repertoire of RA-regulated genes in embryonic tissues is poorly characterized. We performed a comparative analysis of the transcriptomes of murine wild-type and *Retinaldehyde Dehydrogenase 2* null-mutant (*Raldh2* ^−/−^) embryos — unable to synthesize RA from maternally-derived retinol — using Affymetrix DNA microarrays. Transcriptomic changes were analyzed in two embryonic regions: anterior tissues including forebrain and optic vesicle, and posterior (trunk) tissues, at early stages preceding the appearance of overt phenotypic abnormalities. Several genes expected to be downregulated under RA deficiency appeared in the transcriptome data (e.g. *Emx2*, *Foxg1* anteriorly, *Cdx1*, *Hoxa1*, *Rarb* posteriorly), whereas reverse-transcriptase-PCR and in situ hybridization performed for additional selected genes validated the changes identified through microarray analysis. Altogether, the affected genes belonged to numerous molecular pathways and cellular/organismal functions, demonstrating the pleiotropic nature of RA-dependent events. In both tissue samples, genes upregulated were more numerous than those downregulated, probably due to feedback regulatory loops. Bioinformatic analyses highlighted groups (clusters) of genes displaying similar behaviors in mutant tissues, and biological functions most significantly affected (e.g. mTOR, VEGF, ILK signaling in forebrain tissues; pyrimidine and purine metabolism, calcium signaling, one carbon metabolism in posterior tissues). Overall, these data give an overview of the gene expression changes resulting from embryonic RA deficiency, and provide new candidate genes and pathways that may help understanding retinoid-dependent molecular events.

## Introduction

Retinoic acid (RA), an active derivative of the liposoluble vitamin A, is an endogenous signaling molecule involved in many biological processes in vertebrates. This small hydrophobic compound is the ligand for a subfamily of nuclear receptors, the retinoic acid receptors (RAR) α, β and γ (NR1B1, B2 and B3), that belong to the group of nuclear receptors heterodimerizing with RXRs. The RAR-RXR dimers bind to DNA motifs called RA-response elements (RAREs), typically composed of two short similar sequences (“direct repeats”, DR) with a spacer element of varying length (DR1, 2, 5 or 8). Although functional RAREs have yet been characterized in a relatively small number of genes [1], [2], it is estimated that several hundred genes may harbor such elements [3]. RAR/RXR dimers are able to bind RAREs in the absence of ligand and – at least on some of their target promoters –, can have a repressive function by interacting with corepressors that silence transcription through epigenetic mechanisms (ref. [4] for a review). RA binding leads to a conformational change in the receptor structure, leading to a switch from a repressing to a transcriptionally activating state. The distribution and availability of RA in various cell populations therefore has to be tightly controlled by enzymatic mechanisms.

Many of the known functions of RA take place during development. As reported in several species, RA already acts during early embryogenesis, starting at the gastrula stage [5], [6]. Region-specific distributions of RA in embryonic cell populations have been correlated with several developmental events. At early stages, RA may act in combination with other signaling molecules (FGF, Wnts) to provide positional identity along the prospective embryonic anterior-posterior axis (ref. [7] for a review). Once mesodermal segmentation (somitogenesis) is taking place, specific spatial RA distributions are critical for the establishment and patterning of the hindbrain segments (the rhombomeres), whereas more caudally, RA has been implicated in the regulation of the symmetrical progression of somitogenesis, and in early neurogenic events in the neural tube (the prospective spinal cord) [8], [9], [10], [11] (refs. [12], [13], [14] for reviews). Other early embryonic functions have been reported for development of the segmented pharyngeal apparatus (the branchial arches), and in cell populations giving rise to the heart [15], [16], [17], [18].

The distribution of RA among cell populations is controlled by specific enzymatic pathways. Embryos from placental species obtain vitamin A in the form of retinol transferred from the maternal circulation through the embryonic yolk sac – and eventually the placenta [19], [20]. Oviparous species store retinol and/or carotenoids in the egg yolk. Retinol reaching the embryo can be oxidized to retinaldehyde by two classes of enzymes (alcohol and retinol dehydrogenases: ADHs, RDHs), whereas β-carotenoids are cleaved by β-carotene-15,15′ oxygenases (BCOs) (refs. [21], [22], [23], [24] for reviews). Critical for the production of RA is the presence of specific retinaldehyde dehydrogenases (RALDHs). Three such enzymes have been characterized, all of which being expressed according to distinct temporal and spatial (tissue-specific) patterns during development. Interestingly, there is a temporal window during which RALDH2 is the only RALDH to be expressed in the early embryo, and all the functions mentioned above have indeed been ascribed to this enzyme. This phase goes from gastrulation to early somitic stages, during which *Raldh2* gene expression is first induced in the embryonic node and newly formed mesoderm, and then persists at specific anterior-posterior axial levels in several mesodermal derivatives [6], [25]. Analysis of murine *Raldh2* ^−/−^ null mutants harboring a RA-activatable reporter transgene has demonstrated the absence of any detectable RA activity throughout the embryo at these early stages [26]. *Raldh2* expression is transiently seen at early somite stages (E8.0–E8.5 in the mouse) in head tissues, including the anteriormost neuroectoderm destined to form the anterior forebrain and optic vesicles [27], and knockout mutation of *Raldh2* affects morphogenesis of these structures [28], [29]. The second next *Raldh* gene to be expressed is *Raldh3*, whose transcripts first appear by E8.5 in head structures (prospective nasal region, eye, and otocyst), and at trunk levels in the mesonephric duct (embryonic kidney) [30], [31].

**Figure 1:**
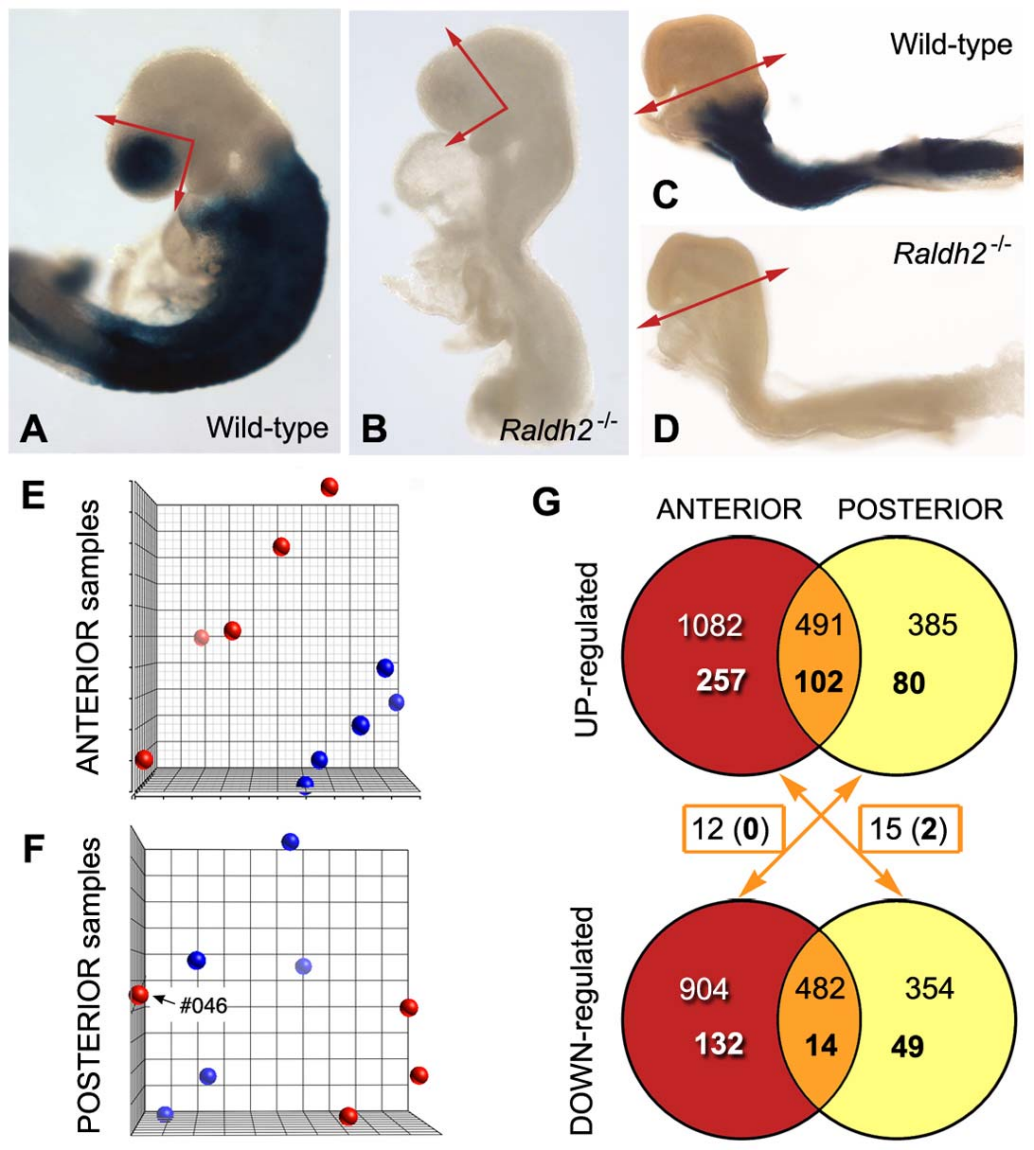
Description of the samples analyzed and summary of the microarray results. (A–D) Profile views of mouse embryos (wild-type: A, C; *Raldh2* ^−/−^ mutants: B, D) at the stages chosen for analysis. The embryos are not from the experimental series, but were collected from litters harboring a retinoic acid-sensitive (RARE-hsp68-*lacZ*) reporter transgene and processed for X-gal analysis, thereby illustrating the absence of detectable RA activity in the mutants. The collected samples were the anterior head region of 14 somite stage embryos (X-gal-labelled in control embryos, section planes illustrated by arrows: A, B) and the posterior region (X-gal-labelled in control embryos, section plane illustrated by arrows: C, D) of 4 somite stage embryos. (E, F) Principal component analysis illustration of ANT and POST data sets. Wild-type samples are depicted by blue dots, and *Raldh2* ^−/−^ samples by red dots. For anterior tissues, wild-type and *Raldh2* ^−/−^ samples segregate in two distinct groups. In the case of posterior tissues, one *Raldh2* ^−/−^ sample (#046) did not cluster with the others, and was excluded from subsequent analyses. (G) VENN diagrams summarizing the numbers of genes showing statistically significant different expression levels, with a fold change of ±1.2 (upper numbers) or ±1.5 (lower, bold numbers). The numbers of genes exhibiting a “contradictory” behavior (upregulated in one type of samples, and downregulated in the other, as pointed out by the arrows) are also given.

Although *Raldh2* ^−/−^ mouse mutants have been used in a large set of studies for unravelling the consequences of a lack of RA signaling in early developmental processes, there is still a poor understanding of the molecular events leading to the realization of the RA-deficiency phenotypes. Genes abnormally expressed in the *Raldh2* ^−/−^ embryos were usually identified through in situ hybridization studies using molecular markers of the affected structures, and there has been no attempt to globally analyze gene expression changes at the transcriptomic level. As this mutant is a good model for analyzing embryos developing under RA deficiency, we decided to perform an Affymetrix DNA microarray study to compare the transcriptomes of wild-type and *Raldh2* ^−/−^ embryos. We searched for gene expression changes in two regions of the embryo: the anterior head and forebrain region, and more posterior (“trunk”) tissues, as these correspond to two distinct – both temporally and spatially – phases of RALDH2-dependent RA signaling. The tissue samples were collected at such stages that RA deficiency was installed in the corresponding region (as documented, for instance, by the analysis of a RA-sensitive transgene; see Fig. 1), although early enough for morphological phenotypic abnormalities to be minimal in the mutant embryos. The resulting data provide a global view on genes affected in their expression by a state of embryonic RA deficiency, and highlight possible gene networks involved in the resulting phenotypic defects, as bioinformatic clustering analysis identified several groups of genes displaying similar behaviors in the mutant tissue samples. More generally, this study confirms the pleiotropic effects of RA signaling in regulating many classes of gene products, some of which belong to critical pathways that will help explain the developmental consequences of a deficiency in this signal.

## Results and Discussion

### Experimental design

Two sets of samples were used for analyzing transcriptome changes in *Raldh2* ^−/−^ embryos. The rostral part of the head (including the anterior forebrain, optic vesicles, and overlying tissues), was collected from wild-type and mutant embryos at the 14 somite stage. This stage was chosen according to the expression profiles of *Raldh* genes in head tissues of wild-type embryos: it shortly follows the phase of peak *Raldh2* mRNA expression at 8-10 somites [27], while RALDH2 protein is still detectable [29], whereas *Raldh3* mRNA expression, first detected at the 10–12 somite stage, has not yet intensified. It is unlikely that RALDH3 significantly participates to RA synthesis in the head at the stage of analysis, and – as described in a previous study [29] – we confirmed by analyzing *Raldh2* ^−/−^ mutants carrying the RARE-hsp68-*lacZ* transgene [32], that there was no detectable activity of the *lacZ* reporter at the 14 somite stage (Fig. 1A, B). The tissues collected for analysis included the optic cup and the forebrain neuroepithelium located more rostrally (prospective telencephalon), as well as the overlying tissues (frontonasal region including the olfactory placode). These samples are referred to thereafter as “anterior” (ANT) tissues.

The posterior tissues were analyzed at the 4 somite stage, and samples were collected from a transverse section plane excluding all tissues from the level of the first branchial arch (Fig. 1C, D). At this stage RA has numerous documented effects (see Introduction), however *Raldh2* ^−/−^ embryos do not yet exhibit morphological deficiencies (see Fig. 1D) – but they can be recognized by trained eyes by the presence of smaller somites and an abnormally shaped heart tube. As expected from previous work [26], there was no detectable activity of the RARE-hsp68-*lacZ* transgene in the mutant embryos at this stage (Fig. 1D). These samples will be referred to as “posterior” (POST).

### Microarray analysis and statistical tests

Total RNA was extracted from individual samples using the RNAeasy micro Kit (Qiagen), and RNA quality was assessed with a 2100 Bioanalyzer (Agilent). Samples that displayed a RNA Integrity Number (RIN) greater than 9.0 were selected for each condition, and 5 mutant and 5 wild-type samples for each tissue type (ANT and POST) were processed on Affymetrix GeneChip Mouse Gene 1.0 ST arrays. The chips were washed and stained in the GeneChip Fluidics Station 450, and scanned with the GeneChip Scanner 3000 7G (Affymetrix). Raw data (.CEL Intensity files) were extracted from the scanned images using the Affymetrix GeneChip Command Console (AGCC) version 3.1. One of the *Raldh2* ^−/−^ POST samples displayed a high background after hybridization and could not be used for analysis.

Altogether, CEL Intensity files from 5 wild-type and 5 *Raldh2* ^−/−^ ANT samples, and 5 wild-type and 4 *Raldh2* ^−/−^ POST samples, were processed for statistical analysis. CEL files were analyzed with the Partek Genomics Suite 6.5 software. After principal component analysis (PCA), the mutant ANT samples clearly segregated from the corresponding wild-type (WT) samples (Fig. 1E), showing relevant transcriptional differences between both groups. Unexpectedly, one of the POST *Raldh2* ^−/−^ samples (#046) did not cluster with the three other mutant samples (Fig. 1F). Statistical studies were performed including or excluding this sample, in order to calculate its impact on the differentially expressed genes. Genes retained for further analysis had a hybridization signal value above 5 (20^th^ percentile of all expression values) in at least one sample. Genes were considered as differentially expressed if the false discovery rate (FDR) from Benjamini and Hochberg test was under 0.1. Among the 35556 probe sets represented in the microarrays, about 10% of the genes were excluded from further analysis because of their low expression level and/or FDR above 0.1 (corresponding to a p-value above 0.05 for ANT samples, or 0.03 for POST samples).

### Overview of differentially expressed genes

Using as a threshold the p-values defined above by Partek analysis, 7177 genes were differentially expressed in wild-type vs. mutant ANT tissue samples (3437 upregulated, 3740 downregulated in mutants; p≤0.05). When POST samples were analyzed by omitting the dubious *Raldh2* ^−/−^ sample (#046, Fig. 1F), 4133 genes were found to be differentially expressed (1834 upregulated, 2299 downregulated in mutants; p≤0.03). When analysis was performed including sample #046, 3120 genes were significantly upregulated (24.5% gene loss), and only 1610 genes appeared downregulated (30% gene loss). Thus, as predicted by PCA, inclusion of sample #046 biased the statistical tests, and data mining was performed with this sample excluded.

**Figure 2:**
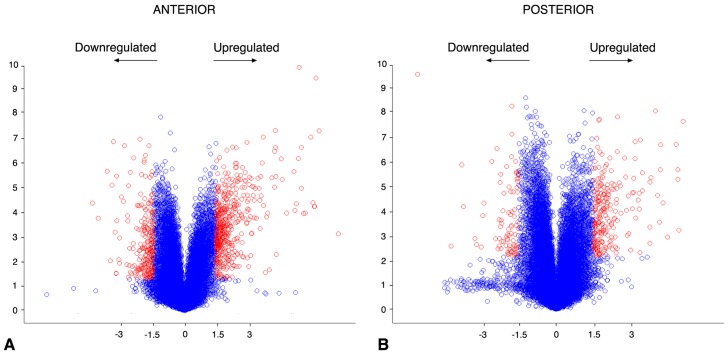
Volcano plots representing corrected p-values (ordinates, −log10 scale) from Student t-test of the mRNA levels compared in wild-type and *Raldh2* ^−/−^ samples anterior (A) and posterior (B) embryonic tissue samples. Genes with a fold change (abscissae) higher than 1.5 and a false discovery rate lower than 0.1 are shown in red.

In both the ANT and POST microarray experiments, the large majority of genes were affected by fold changes (FC) lesser than 2 fold (only 104 genes with FC >2, and 30 genes with FC <−2, in *Raldh2* ^−/−^ vs. wild-type ANT samples; 42 genes with FC >2 and 82 genes with FC <−2, in *Raldh2* ^−/−^ vs. wild-type POST samples). VENN diagrams in Fig. 1G show gene numbers obtained for two cut-offs at ±1.2 FC (upper lines) and ±1.5 FC (lower lines, numbers in bold). The 1.2 FC cut-off yielded comparable numbers of genes up- or downregulated in each experiment, with lower numbers of downregulated genes. Furthermore, there were almost 500 genes upregulated in both the ANT and POST *Raldh2* ^−/−^ tissue samples, and nearly as many genes downregulated in both *Raldh2* ^−/−^ tissue samples. These lists were reduced to 102 genes up-regulated in common, and 14 genes down-regulated in common, at ±1.5 FC. Remarkably, the numbers of “contradictory” genes (being upregulated in one tissue and downregulated in the other) was extremely low (0 and 2 contradictory genes above 1.5 FC; Fig. 1G). Table 1 provides a list of the most severely downregulated genes, and details on contradictory genes are provided as supplementary online information (Table S1). A graphic illustration of the differentially expressed genes is also shown (Fig. 2).

**Table 1:** Most severely downregulated genes in *Raldh2* ^−/−^ tissue samples.

DOWN in ANTERIOR TISSUES (1)		
Symbol	Name	Function
*Id4*	Inhibitor of DNA binding 4	Regulation of transcription and cell proliferation, brain, hippocampus development
*Stat4*	Signal transducer and activator of transcription 4	Regulation of transcription and cell proliferation, signal transduction
*Taf9b*	Taf9b, TBP-associated factor	Regulation of transcription
*Mfsd2*	Major facilitator superfamily domain containing 7B	Unknown
*Car3*	Carbonic anhydrase 3	Lyase activity
*Epha3*	Eph receptor A3	Transmembrane receptor, protein kinase
*Rhag*	Rhesus blood group-associated A glycoprotein	Ammonium transmembrane transporter
*Syt11*	Synaptotagmin XI	Calcium transmembrane transporter
*Ptprz1*	Protein tyrosine phosphatase, receptor type Z, polypeptide 1	Axonogenesis
*Cpa2*	Carboxypeptidase A2	Metallocarboxypeptidase
*Neto2*	Neuropilin (NRP) and tolloid (TLL)-like 2	Membrane receptor
*Gm9983*	Predicted gene 9983	Unknown
*Mfsd7b*	Major facilitator superfamily domain containing 7B	Head, digits, and spleen development
*Fabp7*	Fatty acid binding protein 7, brain	Lipid transport, forebrain neurogenesis
*Slfn9*	Schlafen 9	Unknown
*Jakmip2*	Janus kinase and microtubule interacting protein 2	Kinase, microtubule interacting protein
*Slc4a1*	Solute carrier family 4 (anion exchanger), member 1	Anion exchanger transport
*Nr2e1*	Nuclear receptor 2E1 (Tlx)	Regulation of transcription, brain and eye development
*Idi1*	Isopentenyl-diphosphate delta isomerase	Steroid and carotenoid biosynthesis
*Lonrf2*	LON peptidase N-terminal domain and ring finger 2	Unknown

DOWN in POSTERIOR TISSUES (2)		
Symbol	Name	Function
*Arg1*	Arginase	Arginase activity
*1700011H14Rik*	Unknown	Unknown
*Rarb*	Retinoic acid receptor beta	Transcriptional regulation, embryogenesis, neurogenesis
*Nepn*	Nephrocan	Negative regulation of transcription and of TGFβ receptor signaling
*Hnf1b*	HNF1 homeobox B	Regulation of transcription, inner cell mass differentiation, anterior-posterior patterning, hindbrain development
*Dbx1*	Developing brain homeobox 1	Regulation of transcription, spinal cord interneuron specification
*Rfx6*	Regulatory factor X, 6	Regulation of transcription, pancreatic development
*Pax6*	Paired box gene 6	Regulation of transcription, brain, eye, spinal cord development, neurogenesis
*Hoxa1*	Homeobox gene A1	Regulation of transcription, hindbrain development, anterior-posterior patterning, inner ear morphogenesis
*Nxf3*	Nuclear RNA export factor 3	mRNA export
*Gm10785*	Unknown	Unknown
*Unc5a*	Unc-5 (C. elegans) homolog A	Membrane receptor, apoptosis, signal transduction
*Maob*	Monoamine oxidase B	Oxidoreductase
*Lhx1*	LIM homeobox protein 1	Regulation of transcription, ectoderm/endoderm formation, pattern specification, nervous system and urogenital development
*Slc16a12*	Solute carrier family 16 (monocarboxylic acid transporters), member 12	Symporter activity
*Olig2*	Oligodendrocyte transcription factor 2	Regulation of transcription, oligodendrocyte specification
*Fgfr4*	Fibroblast growth factor receptor 4	Membrane receptor, tyrosine kinase
*Sfrp5*	Secreted frizzled-related sequence protein 5	Extracellular modulator of Wnt signaling, foregut endoderm and mesoderm development
*Cpa1*	Carboxypeptidase A1	Metallopeptidase, proteolysis
*Dhrs3*	Dehydrogenase/reductase (SDR family) 3	Oxidoreductase

DOWN in ANTERIOR and POSTERIOR TISSUES (3)		
Symbol	Name	Function
*Apba2*	Amyloid beta (A4) precursor protein-binding, family A, member 2	Neuronal adaptor protein, synaptic transmission
*Ap1s3*	Adaptor-related protein complex AP-1, sigma 3	Intracellular protein transport, endocytosis
*Ccnb1ip1*	Cyclin B1 interacting protein 1	Blastocyst formation, organ growth, meiosis and spermatid development, apoptosis
*Ccne1*	Cyclin E1	Cyclin-dependent protein kinase, cell cycle control
*Csn3*	Casein kappa	Oocyte meiosis, lactation
*Elovl2*	Elongation of very long fatty acids-like 2	Lipid biosynthesis
*Kbtbd8*	Kelch repeat and BTB (POZ) domain containing 8	Unknown
*Pmm2*	Phosphomannomutase 2	Mannose biosynthesis
*Ptprz1*	Protein tyrosine phosphatase, receptor type Z	Extracellular matrix protein, hydrolase, neuritogenesis
*Rrp12*	Ribosomal RNA processing 12 (S. cerevisiae) homolog	Unknown
*Snhg1*	Small nucleolar RNA host gene	Unknown
*Tmem56*	Transmembrane protein 56	Unknown
*Trmt61a*	tRNA methyltransferase 61 homolog A	tRNA methyltransferase activity
*Pinx1 (2610028A01Rik)*	PIN2/TERF1 interacting, telomerase inhibitor 1	Telomere maintenance, nucleic acid and protein binding

(1) Genes listed by decreasing fold change in expression, ranging from −3.3 to −1.9.

(2) Genes listed by decreasing fold change in expression, ranging from −4.5 to −1.7.

(3) Genes listed alphabetically, fold change in expression <−1.5 in both the ANT and POST microarrays.

The table distinguishes the “top” downregulated genes in anterior or posterior tissues, and the genes affected in both types of samples.

### Down-regulated genes include known RA-dependent genes

As RARs behave as transcriptional activators in the presence of their ligand, down-regulation of retinoid target genes is expected to occur in tissues deficient in RA synthesis. We analyzed the lists of downregulated genes to see whether genes already known to be downregulated in the *Raldh2* mutant, or more generally reported in the literature as being RA-dependent, were detected in our microarray experiments. Several genes shown in previous studies to be downregulated in *Raldh2* ^−/−^ embryos (usually using whole-mount in situ hybridization) [6], [26], [33], [34], [35] were also found downregulated in the POST microarray experiment. These include *Rarb*, *Pax6*, *Hoxa1* (all <−2), *Meox1* (−1.68), and *Cdx1* (−1.40). Additional RA targets were reduced in the POST experiment, although excluded from the filtered list due to their p-values, including *Neurog2* (−1.24) [36], *Olig2* (−1.78) and *Tbx5* (−1.33) [18]. *Stra6*, encoding a membrane protein interacting with retinol-binding protein (RBP) and originally characterized as a RA-induced gene in P19 embryonal carcinoma cells [37], [38] and *Dhrs3* (also known as *RetSDR1*), encoding a retinaldehyde dehydrogenase/reductase, were both reduced in the caudal region. Downregulation of *Dhrs3* may be due to a feedback mechanism caused by retinaldehyde accumulation in embryonic tissues [39]. The *Crabp2* gene (encoding cellular RA-binding protein-II) was downregulated in both posterior and anterior tissues, indicating that the retinoic acid-response elements (RAREs) identified in the *Crabp2* gene promoter [40] are functional targets. The *Nkx3-1* and *Nkx3-2* genes were both reduced in posterior tissue samples. *Nkx3-1* is a RA-inducible target in prostate cancer cells [41].

Several genes previously shown be reduced in the forebrain of *Raldh2* ^−/−^ embryos [29] were also downregulated in our ANT microarray data, for instance *Dll1* (−1.31), *Foxg1* (−1.42) and *Emx2* (−1.56). Thus, a good overall correlation between our transcriptomic data and previous analyses of *Raldh2* ^−/−^ embryos (typically by non quantitative in situ hybridization) confirms the validity and reproducibility of these studies.

### Microarray analysis provides new candidate genes for the RA-deficiency phenotypes

When considering the list of genes exhibiting the highest changes in expression levels in *Raldh2* ^−/−^ embryos (Table 1), some interesting candidates emerged as being potentially involved in the RA-deficiency phenotype. The most highly downregulated genes in anterior tissues encode Id4 (inhibitor of DNA binding 4), a regulator of neural stem cell proliferation and differentiation [42], the signal transducer and activator of transcription Stat4, and the TBP associated factor Taf9b. Among the most severely downregulated genes are also the Ephrin receptor gene *Epha3*, the neuropilin/tolloid-like gene *Neto2*, and the gene encoding fatty acid-binding protein 7 (Fabp7 or brain-type Fabp). The *Nr2e1* gene, encoding the orphan nuclear receptor Tlx, was also strongly downregulated in anterior tissues. A previous study pointed to Tlx as being involved in the regulation of the *Rarb* gene in the eye [43]. The SRY-box genes *Sox1* (−1.66) and, to a lesser extent, *Sox3* (−1.26), were also downregulated according to the ANT microarray experiment.

Retinoids orchestrate the differentiation and patterning of the posterior neural tube (the developing spinal cord) [7], [13], accompanied by induction of a posterior transcriptional signature previously analyzed in the spinal cord at an early fetal stage [44]. Our analysis of posterior embryonic tissues (see Table 1) confirmed many known caudal neuronal targets, including *Pax6*, *Hoxa1*, *Dbx1*, a homeobox gene involved in spinal cord interneuron specification [45] and *Lhx1*, a LIM-homeodomain protein regulating gastrulation, renal organogenesis, and other embryonic functions [46]. Of note, the *Lhx1* homologue *Lhx2* was downregulated in both ANT (−1.60) and POST (−1.45) tissues. Novel downregulated genes included *Sfrp5*, encoding a secreted antagonist of Wnt signaling [47], and *Nepn* (*Nephrocan*), encoding an inhibitor of TGFβ signaling [48]. Other significant reduced targets include *Dmrt2* (doublesex and mab-3 related transcription factor 2), involved in somitogenesis and early myogenesis [49], *Myf5* (a regulator of early myocytic lineage decisions), *Metrn* (encoding Meteorin, a secreted protein controlling neuritogenesis and angiogenesis in glial precursors [50]), *Unc5a* (encoding a Netrin receptor involved in spinal cord development [51]), *Tcf15* (encoding the basic helix-loop-helix protein Paraxis involved in presomitic mesoderm differentiation [52]), and *Rfx6* (encoding a winged helix transcription factor required for pancreatic development [53]).

While a deficiency in embryonic RA should primarily result in lack of activation of RA/RAR inducible genes, feedback mechanisms may lead to upregulation of genes which may also be relevant for the resulting phenotypes. Among the highest upregulated genes were two genes coding for basic helix-loop-helix proteins, *Bhlhe40* and *Bhlhe41* – also known as *Sharp2* and *Sharp1* [54]. *Bhlhe40* was first reported under the name of *Stra13*, as a RA-inducible gene promoting neuronal differentiation in P19 cells [55]. It was therefore unexpected to see it upregulated in vivo in *Raldh2* ^−/−^ embryonic tissues. For this reason we included *Bhlhe40* among the set of genes for which additional analyses (quantitative RT-PCR and in situ hybridization) were performed to confirm the expression changes (see below). Other transcription factor-coding genes were upregulated in *Raldh2* ^−/−^ samples, namely the *Hairy*/*Enhancer-of-Split*-related gene *Hey2*, the winged-helix protein-coding gene *FoxO3*, the zinc finger protein-coding gene *Egr1* (formerly known as *Krox24*), and the orphan nuclear receptor RORα gene (*Rora*). Other upregulated genes included the *Rras* proto-oncogene coding for the R-Ras GTPase, the *Ccng2* gene coding for cyclin G2 – which is regulated at the mRNA level by FoxO3 [56], and the *Vldlr* (very low density lipoprotein receptor) gene, the latter protein also acting as a Reelin receptor during development [57]. Genes encoding ligands or modulators of specific signaling pathways (e.g. *Efna3*, *Fgf11*, *Igfbp1*, *Vegfa*) were also present in the list. A detailed analysis of the main genetic and regulatory pathways emerging from the transcriptomic analysis of RA-deficient embryos is presented below.

### Retinoid targets regulating stem cell lineages

Our transcriptomic analysis has uncovered some novel RA targets regulating neuronal stem cell lineages, and whose deficiency might explain forebrain abnormalities in *Raldh2* ^−/−^ embryos. *Id4, Tlx*, and *Sox1* are all reduced in ANT *Raldh2* ^−/−^ tissues. The mutant embryos exhibit dramatic reductions in telencephalon neuroepithelium growth, a defect previously proposed to be due to reduced fibroblast growth factor (FGF) and Sonic hedgehog signaling [29]. Retinoid regulation of gene targets in stem cells could also stunt cerebral growth. Interestingly, *Id4* regulates lateral expansion of the proliferative zone in the developing cortex [42] by facilitating self-renewal and proliferation of neural stem cells [58]. *Tlx* maintains neural stem cells in an undifferentiated, proliferative state [59], whereas *Sox1*-expression marks activated neural progenitors. Sox1 also functions to maintain cortical progenitors in an undifferentiated state by suppressing cell cycle exit to neurogenesis [60]. Collectively, *Id4, Tlx*, and *Sox1* reductions may be sufficient to affect forebrain development by hindering neural stem cell growth. Neural stem cells may consequently undergo premature differentiation, explaining why we observe increased levels of *Sox10* [29] and *Bhlhe40* in *Raldh2* ^−*/*−^ forebrains. Reduced FGF signaling and cyclin D levels [29] could be a consequence, and not the cause, of reduced progenitor growth. In another study we have shown that *Raldh2* loss of function reduces expansion and differentiation fetal spinal cord-derived neural stem cells [44]. Our ongoing work using neurosphere assays indicates that neural stem cells are similarly affected in the forebrain of 14 somite-stage *Raldh2* ^−*/*−^ embryos (M.P. et al, unpublished data).

Retinoic acid is commonly used as an inducer of neuronal differentiation in various tissue culture cell lines. Remarkably, retinoids can differentiate embryonic stem (ES) cell-derived embryoid bodies to a relatively pure stem cell lineage known as radial glia [61]. Radial glial cells are found at the earliest stages of CNS development and play an important role in cortical expansion. An in vivo role of retinoids in promoting this lineage, though, has never been shown. Two players in the radial glia lineage, *Pax6* and *Fabp7*, are both reduced in ANT *Raldh2* ^−/−^ tissues. Pax6 maintains the proliferative capacity and developmental potential of radial glia stem cells [62], whereas Fabp7 (also known as Brain Lipid Binding Protein, BLBP) is a structural marker also required for the establishment of the radial glia fiber system in the developing brain [63].

### Clustering analysis of gene expression changes in *Raldh2* ^−/−^ embryos

**Figure 3:**
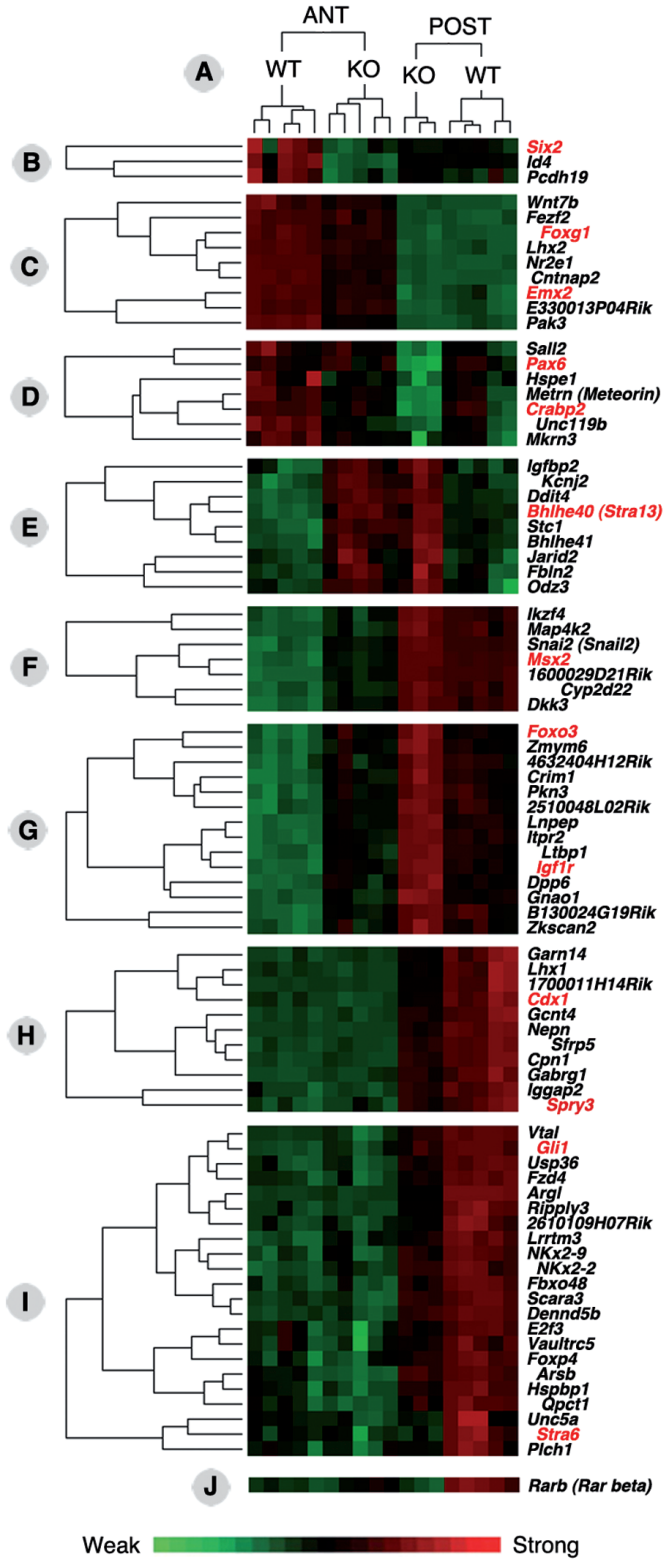
Details of the gene expression profiles obtained after hierarchical clustering of the experimental samples by relative gene expression level analysis. Eight clusters (panels B–I) have been extracted from the overall clustering analysis (available as additional online information, Figure S1), showing differential gene expression behaviors according to the experimental samples (ANT, RNA from anterior tissues; POST, RNA from posterior tissues; WT, wild-type embryos; KO, *Raldh2* ^−/−^ embryos). Gene expression profiles are illustrated as a heat map (green: weak expression; red: strong expression – see scale below). The expression profile of the *Rarb* gene is also shown (panel J), which did not cluster with any other gene. This analysis further validated the experimental samples, as WT and KO samples segregated into fully distinct clusters, both for the ANT and POST tissue samples (panel A above).

To obtain a more global view of the gene expression changes detected in the *Raldh2* ^−/−^ embryos, we performed clustering analysis using the Cluster 3 software [64]. An overview of the entire clustering analysis is provided as online supporting information in the form of a heat map (Figure S1), and selected gene clusters are shown in Figure 3. A green-to-red scale (Fig. 3, bottom) illustrates the gene expression changes between samples, from lowest (bright green) to highest (bright red) relative levels. The clustering analysis further validated our experimental samples, as WT and mutant (KO) samples segregated into distinct clusters, with no co-segregation of any WT and KO sample (Fig. 3A).

Such analysis allows us to pinpoint genes exhibiting similar behaviors (in terms of variation in expression levels) among the analyzed samples. As a first example, the *Id4* and protocadherin 19 (*Pcdh19*) genes clustered with *Six2*, being selectively downregulated in anterior tissue samples (Fig. 3B). Interestingly also, the *Foxg1* and *Emx2* genes – both described as downregulated in the forebrain of *Raldh2* ^−/−^ embryos [29] – were found in the same cluster (Fig. 3C). This cluster also included a Wnt gene (*Wnt7b*), the LIM/homeobox gene *Lhx2*, and the nuclear receptor gene *Nr2e1*. Another interesting cluster contained the *Pax6* and *Crabp2* genes (Fig. 3D). Unlike the former ones, genes from this cluster were downregulated in both anterior and posterior *Raldh2* ^−/−^ tissues samples, while their expression levels were higher in anterior tissues (both in WT and KO situations). Such a behavior is not surprising, as *Pax6* and *Crabp2* are highly expressed in most of the tissue (forebrain and optic vesicle) constituting the ANT samples. The cluster also included *Sall2* (a drosophila *Spalt* homologue), *Unc119b*, *Mkrn3* (*Makorin*), and *Metrn* (*Meteorin*). Another cluster grouped the *Msx2* homeobox gene, a Snail homologue (*Snai2*), a Dickkopf homologue (*Dkk3*), an Ikaros family gene (*Ikzf4*), and the Map kinase gene *Map4k2*, all being relatively mildly downregulated in ANT samples (Fig. 3F).

Genes from the cluster containing *Foxo3* and the IGF receptor gene *Igf1r*, on the other hand, were upregulated in both the ANT and POST *Raldh2* ^−/−^ samples, although globally they were expressed at higher levels posteriorly (Fig. 3G). This 14 gene cluster also contained the chordin-like gene *Crim1*, the latent TGFβ-binding protein gene *Ltbp1*, the zinc finger protein genes *Zkscan2* and *Zmym6*, as well as 3 unannotated Riken gene transcripts.

Two examples of genes exhibiting selective downregulation in the POST *Raldh2* ^−/−^ tissue samples are shown. In the first one, the RARE-containing gene *Cdx1* clusters with *Lhx1*, *Sfrp5*, the nephrocan (*Nepn*) gene, and the gene encoding the FGF pathway inhibitor Sprouty3 (*Spry3*) (Fig. 3H). The second one includes *Gli1*, encoding a transcriptional effector of Hedgehog signaling shown to be downregulated in *Raldh2* ^−/−^ embryos [6]. This gene clusters with *Stra6* and several other potentially interesting genes, including two NKx genes (*NKx2-2* and *2-9*), the Frizzled homologue *Fzd4*, the forkhead protein-coding gene *Foxp4*, *Ripply3* (encoding a Tbx1 repressor), and *Unc5a* (encoding a Netrin receptor).

Finally, the gene encoding RARβ (*Rarb*), a direct RA-inducible gene containing a functional RARE [65] was not included in any gene cluster (Fig. 3J). This behavior could be linked to its weak expression in all anterior tissue samples, whereas downregulation was clearly seen in posterior samples.

### Selected gene analysis by quantitative RT-PCR and in situ hybridization

Some genes were selected to perform real-time quantitative reverse transcriptase-PCR (Q-PCR) analysis in order to validate the changes detected by the microarray experiments. We focused on *Pax6*, expected to be downregulated both in anterior and posterior tissues of *Raldh2* ^−/−^ embryos, *Id4, Six2* and *Stat4* as examples of genes identified by microarray analysis as being downregulated anteriorly, and *Lhx2, Crabp2, Ikzf1* and *Unc5a,* that were scored as being downregulated in both regions of the mutant embryos. We also sought to confirm the upregulation of certain genes identified by microarray analysis, mainly for *Bhlhe40* (*Stra13*) which, as discussed above, was not expected to be upregulated in the RA-deficient embryos. *Creb5* (encoding a cAMP-responsive element binding protein) was chosen as an example of a gene with different behavior in anterior (slightly downregulated) versus posterior (upregulated) tissues.

**Figure 4:**
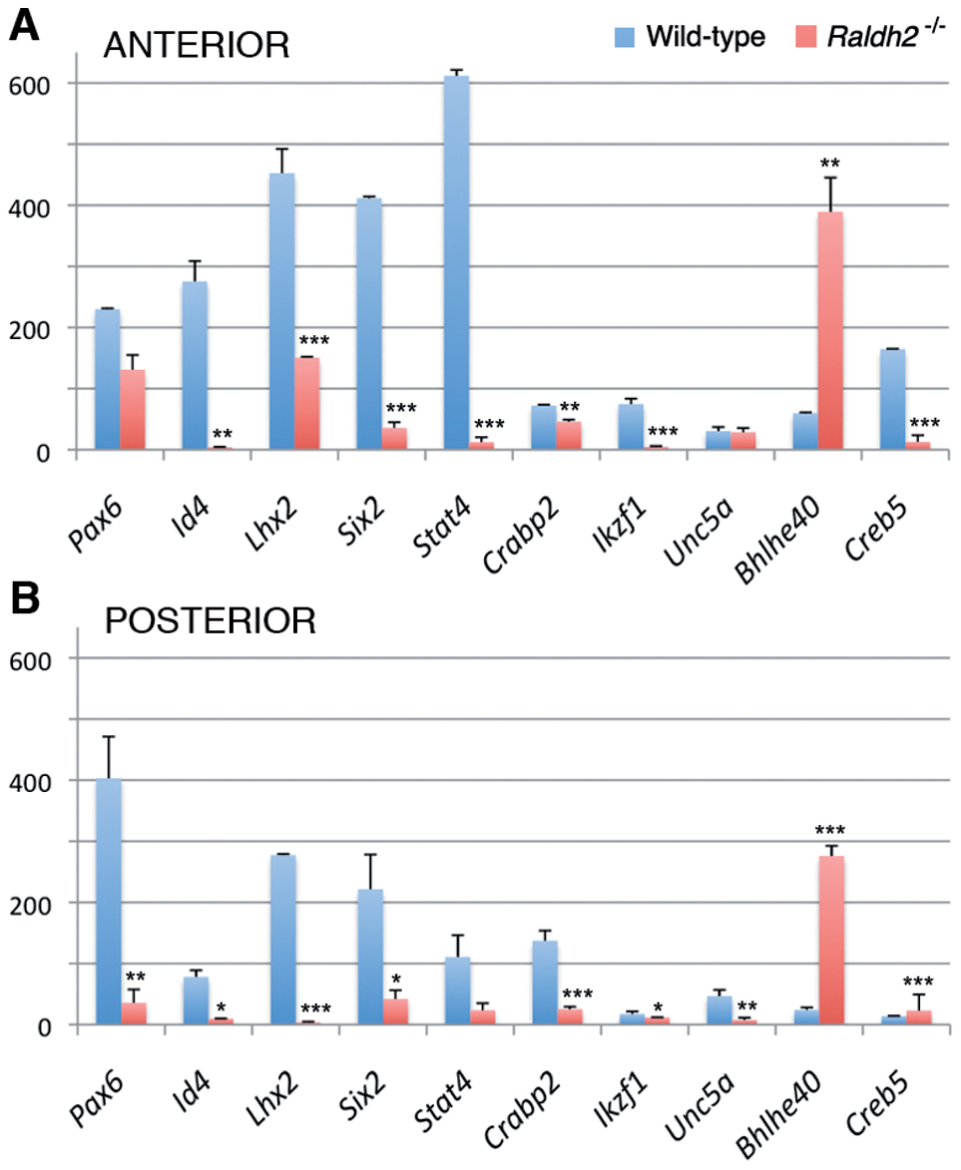
Microarray validation by quantitative real-time RT-PCR (Q-PCR). Genes were selected according to their different expression profiles as detected by microarray analysis of wild-type (WT) and *Raldh2* ^−/−^ embryos. Among these, one gene (*Pax6*) was chosen as a “control”, as its expression had already been shown to be affected in *Raldh2* ^−/−^ embryos (see main text for details and interpretation of the data). Expression levels are expressed as values normalized with respect to *Gapdh* (a housekeeping gene) mRNA levels (WT samples, blue bars; *Raldh2* ^−/−^ samples, red bars). Data (mean ± SEM) were analyzed with Student t-test; ***, p<0.001; **, p<0.01; *, p<0.05.

Overall, the Q-PCR experiments showed a clear correlation with the changes observed by microarray analysis (Fig. 4). Of note, the *Id4* and *Six2* genes, that were scored as downregulated anteriorly in the microarray experiments, were also shown to be downregulated in posterior tissues by Q-PCR analysis, although with a lesser fold change than in anterior tissues. This could be explained by the higher sensitivity of the Q-PCR technique. The same observation was made for *Stat4*, which was downregulated posteriorly – to a lesser extent than in anterior tissues – according to Q-PCR analysis, whereas microarray analysis only revealed an anterior downregulation. In agreement with the microarray data, the *Bhlhe40* gene was markedly upregulated, both in anterior and posterior tissues, according to Q-PCR results. Whereas Q-PCR showed a downregulation of *Creb5* in anterior tissues of *Raldh2* ^−/−^ embryos, there was no significant change in transcript levels in posterior tissues.

Thus, for the selected genes the Q-PCR analysis tended to validate the microarray data. In particular, all genes that were scored as downregulated in the microarray analysis were confirmed by Q-PCR, the latter method often revealing a downregulation in the two types of tissue samples – while microarrays sometimes showed a change only in one type of samples. This points to some limitations in the sensitivity of DNA microarrays, implying that some genes identified as mildly downregulated could in fact be more severely affected in the mutant embryos.

**Figure 5:**
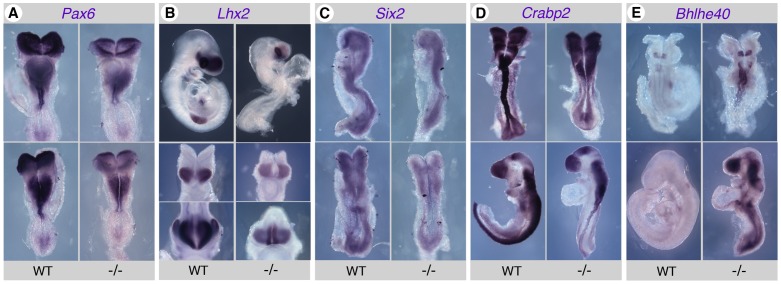
Validation of gene expression changes in *Raldh2* ^−/−^ embryos by in situ hybridization (ISH). A subset of the genes analyzed by Q-PCR yielded detectable signals when performing ISH with antisense riboprobes. Although less sensitive than Q-PCR, this technique allows to analyze mRNA tissue distributions. See main text for an interpretation of the data. Embryos are shown at the 4–6 somite stage for *Pax6* (A: upper panels, ventral views; lower panels, dorsal views), *Lhx2* (B: central panels) and *Six2* (C: upper panels, profile views; lower panels, dorsal views), E9.5 for *Lhx2* (B: upper panels, profile views; lower panels, forebrain frontal views), 6–7 somite stage (upper panels) and early limb bud (lower panels) stages for *Crabp2* (D), 6 somite stage (upper views) and E9.5 (lower views) for *Bhlhe40* (E). WT: wild-type embryos; −/−: *Raldh2* ^−/−^ embryos.

We further attempted to document changes in expression by another technique, in situ hybridization (ISH). These experiments confirmed alterations in transcript levels and/or tissue distribution for some of the genes studied. Consistent with previous reports [29], [34], *Pax6* was downregulated in the caudal neural plate, as well as in more anterior regions of the developing neuroepithelium, including the forebrain, in early somite stage (E8.5) *Raldh2* ^−/−^ embryos (Fig. 5A). *Lhx2* was downregulated in the forebrain of *Raldh2* ^−/−^ embryos, and was undetectable in posterior tissues expressing the gene in control embryos (Fig. 5B). An overall decrease of the *Six2* ISH signal was observed in *Raldh2* ^−/−^ embryos (Fig. 5C). *Crabp2* was downregulated along the developing neural tube and caudal neural plate in early somite stage mutants (Fig. 5D, upper panels), and at later stages was also undetectable in neural crest-derived branchial arches cell populations (Fig. 5D, lower panels). Remarkably, *Bhlhe40* upregulation was also documented in *Raldh2* ^−/−^ embryos by ISH. At early somite stages, higher expression was detected in the hindbrain rhombomeres and extended ectopically along the neural tube (Fig. 5E, upper panels), whereas at later stages an abnormally high expression was observed in most regions of the embryo (Fig. 5E, lower panels). ISH experiments performed with *Id4*, *Stat4*, *Ikzf1* or *Unc5a* probes did not yield any consistent signal, either in wild-type or in *Raldh2* ^−/−^ embryos.

In summary, the changes detected by microarray analysis were for the most part confirmed by Q-PCR for selected genes, and could be documented at the tissue level for some of the genes by ISH. The latter technique has a limited sensitivity, as mRNA detection does not use an amplification step like in RT-PCR, and its readout is qualitative rather than quantitative. It is therefore not unexpected that some gene expression changes documented by transcriptome analysis and Q-PCR may not be visualized through ISH.

### Bioinformatic analysis of the transcriptomic data

**Figure 6:**
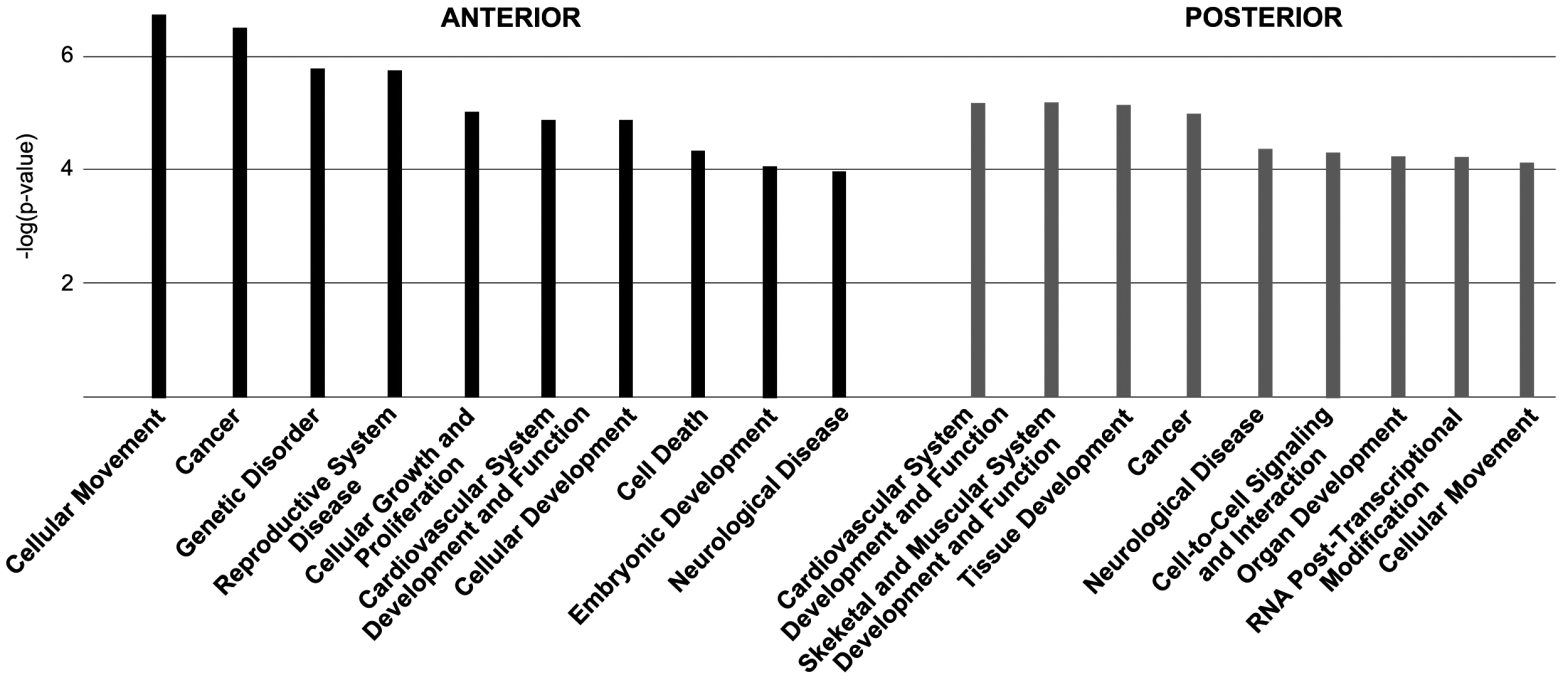
Overview of the main biological and physiological functions correlating with the affected genes in ***Raldh2*** **^−/−^ embryos.** The functions are listed by decreasing order of statistical significance [−log(p-values)] of differentially expressed and misregulated genes as calculated by the Ingenuity software. Left side (black bars): ANT microarray data; right side (gray bars): POST microarray data.

To get further insight into the cellular functions and classes of molecules that may be affected by RA deficiency, we performed an overall analysis of the transcriptome alterations using the Ingenuity Pathway Analysis (IPA) software. Figure 6 gives an overview of the main biological functions (with the more significant and therefore low p-values according to Ingenuity analysis) associated with the transcriptome of *Raldh2* ^−/−^ embryos. These functions are clearly relevant as they relate to general developmental processes (Cellular Development, Embryonic Development, Organ Development), or more specific phenomenons for which retinoid signaling has already been implicated (Cardiovascular System Development, Skeletal and Muscular System Development for the POST microarray experiment). Inclusion of more basic biological functions (Cell-to-Cell Signaling and Interaction, Cellular Movement, Cellular Growth and Proliferation), as well as disease-related categories (Cancer, Neurological Disease, Genetic Disorder, Reproductive System Disease) are also not surprising considering the known effects of retinoids on cell growth and their use in clinical trials for chemotherapy or prevention of certain cancers [66]. These observations prompted us to further analyze the types of molecules and the molecular pathways affected by embryonic RA deficiency.

**Figure 7:**
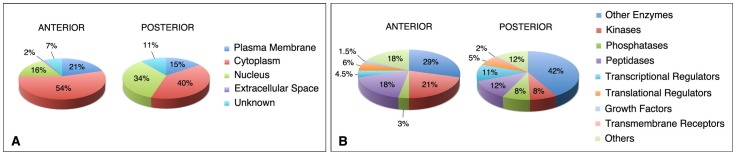
Main classes of molecules downregulated in *Raldh2* ^−/−^ embryonic tissues, according to Ingenuity pathway analysis of the transcriptomic data. Pie charts illustrate the distribution of gene products according to subcellular location (A) or protein function (B), when considering the overall set of affected genes (fold change ±1.2, filtered for FDR <10% and a p-value ≤0.03 for POST and ≤0.05 for ANT).

When the transcriptome data were sorted according to the subcellular localization of the corresponding proteins, there was an over-representation of nuclear proteins (mainly at the expense of cytoplasmic proteins) among genes affected in the POST microarray experiment (Fig. 7A). All major classes of molecules were represented among the sets of affected genes, both for the ANT and POST experiments (Fig. 7B). Notably, there was an over-representation of transcriptional regulators among affected genes in the POST experiment, whereas translational regulators were under-represented when compared to the ANT data set. A list of genes/proteins involved in transcriptional regulation and identified as down-regulated in the ANT and POST microarray experiments is provided as supplementary online information (Table S2).

### Molecular pathways affected in *Raldh2* ^−/−^ embryos

**Figure 8:**
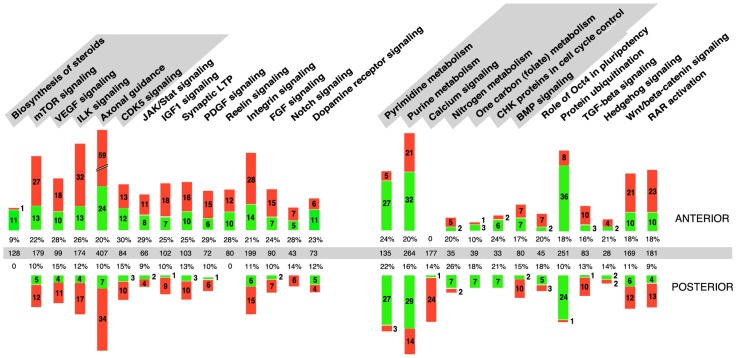
Summary diagram of the major molecular pathways emerging from Ingenuity analysis of the *Raldh2* ^−/−^ transcriptome. The molecular pathways are listed by decreasing order of statistical significance as they appear through analysis of the ANTERIOR (left-side list) and POSTERIOR (right-side list) data sets. The most significant pathways identified for each data set are highlighted in gray. Additional pathways relevant for developmental processes are also listed. A graphic representation of the numbers of genes downregulated (green) or upregulated (red) in anterior (upper bars) or posterior (lower bars) *Raldh2* ^−/−^ embryonic tissue samples (fold change ±1.2, filtered for FDR <10%) is shown. The total number of genes comprising each pathway (middle line, gray shaded), and the percentages of genes misregulated in each experiment, are also given.

Alteration of retinoic acid signaling has already been shown to interfere with several signaling pathways within the embryo (refs. [22], [67], [68] for reviews). Often the changes were described as changes in expression levels of ligands, such as FGFs [8], [9], Wnts [69] or TGFβ [70]. In the case of the Sonic hedgehog (Shh) pathway, no change in ligand expression was observed in head or posterior embryonic tissues of *Raldh2* ^−/−^ mutant embryos, although expression of effectors of this pathway was altered [6], [29]. Analysis of the transcriptomic data provides a more global overview of the molecular pathways that may be affected under conditions of embryonic RA deficiency. A graphic summary of the main pathways identified by Ingenuity analysis is provided in Figure 8. The molecular pathways are listed by decreasing order of statistical significance as they appear through analysis of the ANT (left-side list) and POST (right-side list) data sets. The six “top” (most significant) pathways for each data set are highlighted in gray, and additional pathways relevant for development are also illustrated. The numbers of genes downregulated (green bars) or upregulated (red bars) are illustrated (upper bars: ANT transcriptomic data; lower bars: POST transcriptomic data).

As observed through the analysis of biological functions, several “top” pathways emerging from the ANT microarray data are related to nervous system development or function (axonal guidance, Reelin signaling, Notch signaling, synaptic long term potentiation, dopamine receptor signaling). Although some of these phenomenons take place at later developmental stages than those analyzed, their presence in the top list is easily explained as they contain genes also acting during early embryogenesis. The main molecular pathways identified by the analysis of POST microarray data are more varied, encompassing categories with large numbers of molecules (pyrimidine metabolism, purine metabolism, protein ubiquitination), and more specific pathways with a highly significant p-value, but with much fewer misregulated genes. For instance, the “role of Oct4 in pluripotency” pathway appeared among the top pathways with only 5 downregulated genes (*Foxa1*, *Foxd3*, *Hoxb1*, prohibitin *Phb*, and Polycomb group ring finger *Pcgf6*). The “calcium signalling” pathway was characterized by the presence of 24 upregulated (vs. 1 downregulated) genes, including those encoding alpha 1 (skeletal muscle) actin (*Acta1*), cardiac myosin light chain 3 (*Myl3*) and heavy chain 7 (*Myh7*), calcium/calmodulin-dependent protein kinase IG (*Camk1g*), Creb5, Nfatc4, Nfat5, and the histone deacetylase genes *Hdac5* and *Hdac8*. Inclusion of the “one carbon (folate) metabolism” pathway among the most significantly affected ones may reveal a novel retinoid function, so far poorly studied, although some evidence for a regulation of this pathway by retinoids emerges from metabolomic studies in embryonic stem cells (L.J. Gudas, personal communication). The downregulated genes include *Dhfr* (dihydrofolate reductase) and *Mthf1* (methylene tetrahydrofolate dehydrogenase). Furthermore, the folate receptor-coding gene *Folr1* was markedly downregulated (−2.35), although with a p-value >0.05. Another potentially interesting pathway is that relating to CHK proteins in cell cycle control, which contained 7 downregulated genes, including *Cdc25a*, genes coding for E2F transcription factors 3 and 4 (*E2f3*, *E2f4*), Replication factor C3 (*Rpc3*), and Replication protein A1 (*Rpa1*).

Many of the signaling pathways acting during early embryogenesis appeared with a high significance value in the bioinformatic analysis of the transcriptomic data. This supports the idea of complex RA-dependent regulatory events, and “cross-talks” between the retinoid and other embryonic signaling pathways. Interestingly, among the top (most significant) pathways listed for the ANT transcriptomic data, two were not the major candidates that would be predicted from the literature: VEGF signaling and ILK signaling. Three *Vegf* genes (*Vegfa*, *Vegfb*, *Vegfc*), as well as the Platelet-derived growth factor C (*Pdgfc*) and Placental growth factor (*Pgf*) genes, appeared among the genes upregulated in both pathways (see Table 2 for a full list of misregulated genes in the VEGF signaling pathway). CDK5 and JAK/Stat signaling were also among the highest significant pathways in ANT tissues. Most affected genes in this category also appear as effectors in other pathways (see Table S2 for details). It is noteworthy to mention *Stat4* as markedly downregulated (−3.07), *Stat1* more mildly downregulated (−1.24), and *Stat2*, *Stat5b* and *Stat6* upregulated in ANT tissues of mutants.

**Table 2:** Genes acting in selected signaling pathways and misregulated in *Raldh2* ^−/−^ tissue samples (ANT: anterior samples; POST: posterior samples).

PATHWAY		ANT	ANT	POST	POST
		Upregulated	Downregulated	Upregulated	Downregulated
**FGF**	Ligands	**Fgf10, Fgf11, Fgf16**	Fgf13, Fgf17	Fgf10, **Fgf11**	
	Receptors	**Fgfr2**			***Fgfr4***
	Effectors	Akt3, Gab1, Map2k1, Map2k6, Map3k1, Mapk3, Mapk13, **Met**, Plcg1, Rac3	Hras, Mapk11, Pik3c2B, Pik3cb, **Pik3r3**	Akt3, Frs2, Map3k1, **Creb5**	Mapk13, Hras
**BMP**	Ligands	*Bmp2*, **Bmp4**		Bmp2, Bmp4, Bmp5	
	Receptors		Bmpr1b	Bmpr2	Bmpr1b
	Effectors/Modulators	**Id1, Fst**, Fstl3, **Gpc3**, Grem1, **Htra1**, Map2k1, Mapk3, **Mapk13**, **Msx2**, Pitx1, Pitx2, **Rras**, Smad7	Ecsit, Hras, Mapk10, Mapk11, Mras, Prkag1, Prkag2, Prkar1b	Mapk10, Msx2, **Pitx1**, Pitx2, Prkar1b, **Rras**, Smad7	Hras, Mapk13
**TGFβ**	Ligands	Inha, *Tgfb1*		Inha, *Tgfb1*, **Tgfb2,**	
	Receptors	Acvr2a, Ptgfr, Tgfbr2, Tgfbr3		Tgfbr2, *Tgfbr3*	
	Effectors/Modulators	**Ltbp1**, Map2k1, Map4k1, Mapk3, **Rras**, *Smad6*, Smad7, **Tgfb1i1, Tgfbi**, Tgif1	**Foxh1**, Hras, Mras, Stat1	Eng, Ltbp1, **Rras**, Serpine1, Smad7, **Tgfbi, Thbs1**	Foxh1, Hras, Tgfbrap1
**Hedgehog**	Ligands				***Ihh***
	Receptors	Ptch1			Ptch2
	Effectors	Dyrk1b, Prkar1b, **Hhip**	Prkag2	Prkar1b, Dyrk1b	Gli1
**IGF**	Ligands	Ctgf, Cyr61			
	Receptors	Igf1r		Igf1r	
	Effectors/Modulators	Akt3, Igfbp2, **Igfbp3**, Igfbp4, Igfbp5, Irs1, **Foxo3**, Grb10, Map2k1, Mapk3, Prkar1b, Rac3, **Rras**, Shc1	Hras, Pik3c2B, Pik3cb, **Pik3r3**, Prkag1, Prkag2	Akt3, **Foxo3, Igfbp2, Igfbp3**, Igfbp5, Prkar1b, **Rras**, Shc1	Hras
**VEGF**	Ligands	Figf, Pgf, **Vegfa**, Vegfb, Vegfc		Pgf, **Vegfa**, Vegfc	
	Receptors	Flt1, Flt4		Flt1	
	Effectors/Modulators	**Actc1, Actg2**, Actn3, Akt3, **Foxo3**, Map2k1, Mapk3, Plcg1, Rac3, **Rras**, Shc1	Eifay, Eif2b1, Eif2b4, Hif1a, Hras, Mras, Pik3c2B, Pik3cb, **Pik3r3**	Acta1, Actn2, Akt3, **Foxo3**, Kdr, **Rras**, Shc1	Eif2b1, Eif2b4, Eif2s2, Hras
**Wnt**	Ligands	*Wnt1*,	**Wnt7a**, Wnt7b	*Wnt2*, *Wnt4*, Wnt5a	*Wnt7a*
	Receptors	Acvr2a, Cd44, *Fzd5*, Fzd7	Fzd9, **Ldlr**	Cd44, *Frzb, Ldlr*	Fzd4,
	Effectors/Modulators	*Aes*, Akt3, **Cdh1, Cdh5, Dact1,** **Dkk1**, Dkk3, **Sostdc1, Calcoco1**, Hbp1, Kremen1, Lrp1, Lrp5, Mitf, Porcn, Pp2r5c, Ppp2r3a, Ppp2r5c, Prkcd, Rac3, Ror2	Apc2, *Celsr2*, Ndp, Ppp2r1b, Ppp2r2c,	Akt3, **Calcoco1**, Cdh3, Dakt1, Dkk1, Dkk2, Gnao1, Hbp1, Lrp1, Mitf, Ppp2r3a, Rspo1, Rspo3, ***Sostdc1***, Tcf3, Wisp1	Hnf1a, ***Lect2*** *, Ndp*, Ppp2r1b, **Sfrp5**
**Retinoic acid**	Synthesis enzymes	**Aldh1a2,** **Aldh1a3, Aldh1a7**	Rdh10		*Adh1*
	Metabolism enzymes			Cyp26c1	*Cyp26b1*
	Receptors	Rara, Rarg		Rarg	**Rarb**
	Effectors/Modulators	Cited2, Ncoa1, Nr2f1 (Coup-Tf1), Nr2f2 (Coup-Tf2), Pnrc1, **Vegfa**	Crabp2, Gdap1, Gdap2	Ncoa1, Pnrc1, Rarres2, **Smarca2, Vegfa**	**Crabp2, Raet1d**, Rbp1, ***Rbp4***, **Stra6, Triml2, ** ***Ttr***

Key: **Bold** indicates a fold change exceeding ±1.5.

*Italics* indicate a p-value >0.05 (ANT) or >0.03 (POST).

Table 2 gives details on genes misregulated belonging to several major embryonic signaling pathways (FGF, BMP, TGFβ, Hedgehog, IGF, Wnt/beta-catenin, and the retinoid pathway itself). In most cases the affected genes encoded for a subset of the ligands, more exceptionally a receptor (*Fgfr2* upregulated in ANT tissues, *Bmpr1b* and *Bmpr2*, respectively downregulated and upregulated in POST tissues), and various effectors of the pathway (including *Mapk* genes acting in several pathways). While several *Fgf* genes were upregulated (Table 2), *Fgf8*, previously shown to be upregulated in RA-deficient embryos [8], [9], [10] was not picked up by the microarray analysis, an explanation being that the shift in the *Fgf8* mRNA gradient [71] only occurs in a small region of the *Raldh2* ^−/−^ embryos (the anteriormost presomitic mesoderm). Upregulation of several *Bmp* genes – the highest for *Bmp4* in ANT tissues, is also noteworthy. Within the TGFβ pathway there were a majority of upregulated genes, both in ANT and POST tissues. This is consistent with a previous study which identified the TGFβ pathway as being abnormally upregulated and involved in the phenotypic abnormalities observed during early embryonic lung development [70]. The Hedgehog signaling pathway exhibited the smallest numbers of misregulated genes (6 in ANT tissues, 4 in POST tissues), reflecting the small number of genes (28) included in this category, and included *Gli1* (previously identified as downregulated in *Raldh2* ^−/−^ embryos: ref. [6]) and Indian hedgehog (*Ihh*, downregulated in POST tissues).

In summary, this bioinformatic analysis confirmed that retinoic acid deficiency affects the regulation of genes involved in a wide range of molecular pathways. Remarkably, the main developmental signaling pathways previously implicated as being retinoid-dependent (FGF, TGFβ/BMP, Hedgehog, Wnt; refs. [22], [67], [68] for reviews) do not appear as the top pathways in terms of statistical significance, hence the analysis summarized in Fig. 8 provide new information on metabolic processes or signaling pathways that may deserve further investigation.

### Cross-comparison of in vivo (embryonic) data with results from genomic and transcriptomic screens in embryonic stem cells

Several recent studies have used differentiating murine ES cells as a model for analyzing transcriptomic changes occurring after RA treatment [72], [73], [74], and/or for analysis of genomic loci bound by RARs, using large-scale chromatin immunoprecipitation (ChIP-ChIP or ChIP-seq) techniques [72], [75], [76]. We decided to perform a cross comparison of such studies, to identify which, among the genes downregulated in *Raldh2* ^−/−^ embryos, may also have been identified as being RA-inducible, or RAR-bound according to ChIP data, in differentiating ES cells. This comparison was performed with the data of Moutier et al., who reported the most extensive set of RAR-bound loci, identifying 6628 RAR occupied loci in ES cells grown for 4 days as embryoid bodies (EBs) and treated for 2 h with RA [72]. To narrow down the cross-comparison, their genomic list was restricted to loci in which the RAR-occupied sites were located within ±5 kilobases (Kb) from the transcription start site. These authors also performed a comparative transcriptomic (RNA-seq) analysis of EBs grown for 4 days and treated (or not treated) with RA, identifying 824 RA-induced transcripts [72]. We further used the data of Kim et al., who identified by Affymetrix array analysis 1202 gene transcripts upregulated in ES cells driven to a neural phenotype after exposure to RA from day 4 to 8 in a 12-day suspension culture [73]. Unekama et al. also reported transcriptional signatures of EBs driven to neural differentiation, and here we used their data set of 147 genes induced at day 8 of differentiation, after RA treatment from day 2 to 6 [74].

**Figure 9:**
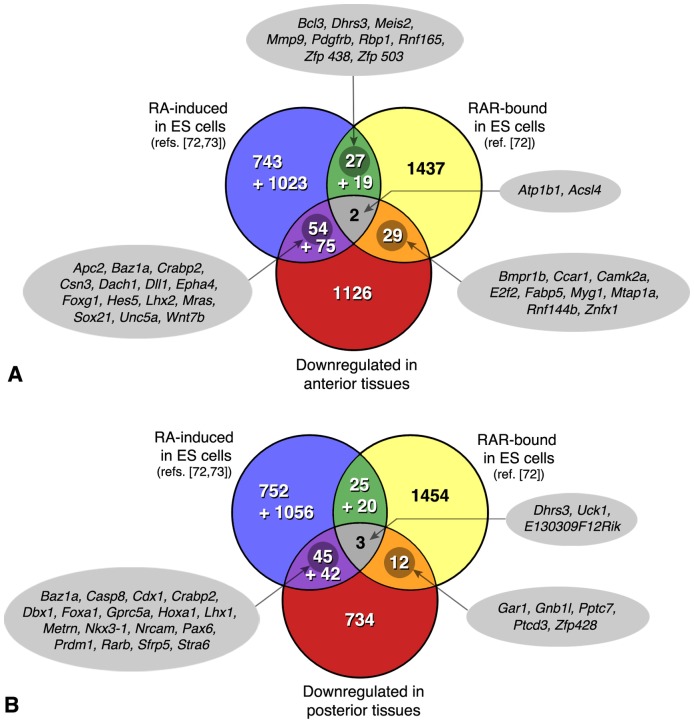
VENN diagrams summarizing a cross-comparison of the genes downregulated in *Raldh2* ^−/−^ embryos (A, anterior tissues; B, posterior tissues) with those identified as RAR-bound by ChIP-seq analysis of embryonic stem (ES) cells differentiating as embryoid bodies (ref. [**72**] **), and those induced after RA treatment of embryoid bodies.** This analysis distinguished early RA-responsive genes (2 h after RA exposure: upper numbers; ref. [72]), from those identified at a later stage of differentiation (4 days post-RA treatment: lower numbers; ref. [73]). Some selected genes are highlighted in the intersecting data sets.

Figure 9 provides an overview of this comparative analysis in the form of Venn diagrams. An immediate major conclusion is that there are very limited overlaps between the sets of genes identified in this study, and those described as RA-inducible or RAR-bound in ES cells. Indeed there were only 31 (∼2.2%) of the genes downregulated in anterior tissues, and 15 (∼1.8%) of those downregulated in posterior tissues of *Raldh2* ^−/−^ embryos, that were tagged as RAR-bound by Moutier et al. [72]. Also, only 4% of the “anteriorly-affected” genes, and 5.5% of the “posteriorly-affected” genes appeared in the RNA-seq data of Moutier et al., i.e. behave as early RA-inducible genes in EBs. Additional genes were found by comparing our data to those of Kim et al. [73] and Akanuma et al. [74], which correspond to transcriptional signatures at a later stage of neural differentiation (2–4 days after the RA treatment). The complete gene lists are available as online supporting information (Table S3).

Surprisingly, only a handful (2 and 3 genes, respectively) were common to the three data sets, i.e. were RA-inducible and RAR-bound in ES cells, and affected anteriorly or posteriorly in *Raldh2* ^−/−^ embryos (Fig. 9). Whereas the anteriorly affected genes did not obviously appear as “developmental” genes (*Atp1b1*, ATPase, Na/K transporter β1 polypeptide; *Acsl4*, acyl-coA synthetase family member 4), the retinaldehyde dehydrogenase/reductase gene *Dhrs3* [39] was one such gene affected posteriorly. It may indeed be a key gene for feedback mechanisms regulating retinoid levels, both in the embryo and in cell systems. The few other genes common between our lists and the RAR-bound loci included several interesting candidates, namely the gene encoding fatty acid-binding protein (Fabp) 5 – identified in other cell systems as a regulator of the anti-apoptotic and proliferative response to RA [77]), or genes encoding the BMP receptor Bmpr1b, the cell cycle regulator Ccar1, and zinc finger (Znfx1, Zfp428) or ring finger (Rnf144b) proteins. Examples of genes downregulated in *Raldh2* ^−/−^ embryos, and reported as early RA-responsive in ES cells [72], are given in Fig. 9. While these include several known RA-target genes (described above), they highlight additional genes whose misregulation may be relevant for the RA-deficiency phenotypes.

### Analysis of *cis*-regulatory elements in downregulated genes

Lastly, we used another bioinformatic approach to assess whether the genes defined here as being RA-dependent in the early embryo, may share other common regulatory features. We used the oPOSSUM online resource and database (refs. [78], [79]; http://opossum.cisreg.ca/oPOSSUM3/) to perform this analysis. The oPOSSUM single site analysis (SSA) tool allows to identify over-represented transcription factor binding sites (TFBS) in specific sets of genes, giving scores to assess over-representation either at the gene level, or at the TFBS occurrence level. We interrogated the oPOSSUM database for SSA analysis on two sets of genes, corresponding to the most severely downregulated genes (FC <−1.5) in anterior or posterior *Raldh2* ^−/−^ tissues (146 and 63 genes, respectively). A summary of the retrieved data is provided in Table 3. Interestingly, the first ranked transcription factor (highest Z-score) for posteriorly downregulated genes is the RAR:RXR DR5 dimer. While this result further tends to validate our transcriptomic data, the corresponding gene hits were only 8, which included *Dhrs3*, *Meox1*, *Metrn*, *Rarb*, and *Stra6*. This finding would suggest that only a small fraction of the genes identified as downregulated in our study, are actually direct targets of RAR:RXR regulation.

**Table 3:** Bioinformatic analysis of putative transcription factor binding sites (oPOSSUM3 single site analysis method) in genes showing the highest downregulation in posterior or anterior tissues of *Raldh2* ^−/−^ embryos.

Transcription factor (c)	Family	Gene hits	TFBS hits	Z-score	Transcription factor (c)	Family	Gene hits	TFBS hits	Z-score
**RXR:RAR (DR5)** (d)	Nuclear receptor	8	11	11.40	**Nobox**	Homeodomain	107	679	21.90
**MZF1_5-13**	ββα zinc finger	43	272	10.27	**NKx2-5**	Homeodomain	124	1566	19.22
**Nobox**	Homeodomain	48	262	9.90	**Pdx1**	Homeodomain	120	1115	17.73
**Foxa1**	Forkhead	40	222	8.14	**Hoxa5**	Homeodomain	123	1574	15.15
**Foxa2**	Forkhead	40	152	7.84	**REST** (l)	ββα zinc finger	6	6	14.66
**Pax5** (e)	Paired domain	8	8	7.78	**Prrx2**	Homeodomain	109	938	13.98
**SRY**	HMG group	48	328	6.87	**Sox9**	HMG group	88	379	12.99
**NKx2-5**	Homeodomain	52	606	6.86	**SRY**	HMG group	112	782	10.28
**HNF1B**	Homeodomain	17	27	6.58	**Sox17**	HMG group	109	558	10.16
**ARID3A**	Arid	48	494	6.08	**NFYA**	NFY CCAAT binding	51	91	10.02
NR2F1 (f) (COUP-TF I)	Nuclear receptor	17	23	5.74	Sox5	HMG group	99	554	8.86
STAT1 (g)	Stat	19	40	5.49	Foxd1	Forkhead	93	496	7.78
HNF1A	Homeodomain	14	16	5.36	NKx3-1	Homeodomain	103	530	6.63
Pdx1	Homeodomain	49	418	5.35	Foxd3	Forkhead	85	340	5.86
PBX1 (h)	Homeodomain	15	25	5.21	Pou5f1 (Oct4) (m)	Homeodomain	26	40	5.81
PPARG:RXRA (i)	Nuclear receptor	22	40	3.96	Lhx3	Homeodomain	41	88	5.56
Hoxa5	Homeodomain	52	607	3.70	Foxo3	Forkhead	95	537	5.47
T (Brachyury) (j)	β-ribbon	8	10	3.63	NR3C1 (GR) (n)	Nuclear receptor	21	28	5.12
STAT3	Stat	32	82	3.48	HNF1B	Homeodomain	33	54	4.70
Pax6 (k)	Paired domain	5	6	3.26	Foxa2	Forkhead	82	323	3.97

(a) 59 genes analyzed from 63 genes downregulated with FC <−1.5.

(b) 137 genes analyzed from 146 genes downregulated with FC <−1.5.

(c) The searched region encompassed 5 Kb upstream and 5 Kb downstream of the transcription start site. The top ten factors (highest Z-scores) appear in bold. Additional transcription factors (below) are the authors' seelection.

(d) Genes with conserved RAR:RXR (DR5) binding sites are: *Csn3, Dhrs3, Fap, Meox1, Metrn, Rarb, Stra6, 5730596B20Rik* (NB: *5730596B20Rik* is an antisense EST found between the *Hoxa3* and *Hoxa4* genes).

(e) Genes with conserved Pax5 binding sites are: *Apba2, Crabp2, Lgr5, Mettl1, NKx2-9, Pmm2, Ripply3, Rfx6*.

(f) Genes with conserved NR2F1 binding sites are: *Apba2, Crabp2, Dhrs3, Dusp9, Fap, Gcsh, Hoxa1, Maob, NKx2-9, Pax6, Prdm13, Rarb, Sfrp5, Stra6, Timm8a1, Tmem56, 1700011H14Rik.*

(g) Genes with conserved STAT1 binding sites are: *Apba2, Dbx1, Dhrs3, Fap, Hoxa1, Gcsh, Kynu, Lgr5, Meox1, Pax6, Prdm13, Ptprz1, Rarb, Ripply3, Slc25a10, Snora34, Stra6, Timm8a1, 5730596B20Rik.*

(h) Genes with conserved PBX1 binding sites are: *Ccne1, Dbx1, Fap, Hoxa1, Kynu, Lgr5, Nepn, Nkx2-9, Nkx3-1, Pax6, Prdm13, Rarb, Rfx6, Ripply3, Tmem56.*

(i) Genes with conserved PPARG:RXRA binding sites are: *Apba2, Cpn1, Crabp2, Dbx1, Dhrs3, Dusp9, Gcsh, Hoxa1, Lhx1, Meox1, Mtap7d2, Nkx3-1, Pax6, Prdm13, Ptprz1, Rarb, Sh3bgrl2, Snord118, Stra6, Trmt61a, 1700011H14Rik, 5730596B20Rik.*

(j) Genes with conserved T binding sites are: *Lhx1, NKx2-9, Pmm2, Prdm13, Stra6, Timm8a1, Trmt61a, 5730596B20Rik.*

(k) Genes with conserved Pax6 binding sites are: *Dbx1, Pax6, Rarb, Stra6, 5730596B20Rik.*

(l) Genes with conserved REST binding sites are: *Acsl6, Ank1, Cntnap2, Lhx2, Mpped1.*

(m) Genes with conserved Pou5fI binding sites are: *Abcb10, Acsl6, Ank1, Apba2, Arrdc4, Aven, Cdh20, Cntnap2, Dhrs11, Gas5, Htr3b, Ikzf1, Lhx2, Mrpl18, Muc1, Neto2, Nova1, Nr2e1, Pak3, Pcdh19, Prdm16, Rragb, Shox2, Slc25a21, Taf1d, Wnt7a.*

(n) Genes with conserved NR3C1 binding sites are: *Cntnap2, Eef1d, Fez1, Hsd17b7, Htr3b, Itgb8, Klf1, Lrcc4, Lhx2, Mpped1, Mrpl20, Nova1, Nsdhl, Pcdh19, Pdss1, Phyhipl, Pou3f3, Rnf144b, Shox2, Syt11, AI504432.*

**Transcription factor binding site (TFBS) analysis: genes downregulated in POSTERIOR tissues.^(a)^**

**TFBS analysis: genes downregulated in ANTERIOR tissues.^(b)^**

Among other transcription factors with overrepresented binding sites are several forkhead factors (Foxa1, Foxa2 posteriorly, Foxa1, Foxd1, Foxd3, Foxo3 anteriorly). Binding sites for high mobility group (SRY-related or Sox) factors were also amongst the most overrepresented. Unlike RAR:RXR binding sites, these yielded very high numbers of hits, both in terms of gene numbers (in all cases >50% of the genes analyzed) and of TFBS identified (up to 782 per gene; see Table 3). This abundance probably reflects an overinflation of putative TFBS, often encountered with bioinformatic search engines, when the TFBS consensus is defined as a relatively short and degenerate sequence. The same conclusion was drawn for most of the homeodomain proteins identified in this search (e.g. Nobox, NKx2.5, Pdx1 or Hoxa5, all of which appeared with high Z-scores for both the anterior and posterior gene sets, and yield up to >1000 TFBS hits). This clearly points to a limitation of the search engine. However, and despite this overinflation, we would conclude that TFBS bioinformatic analysis will help selecting subsets of *Raldh2* ^−/−^ misregulated genes that may be regulated by specific homeodomain factors. A possible, combinatorial regulation by forkhead (Fox) and Sox proteins will also deserve further investigation.

On the other hand, the TFBS analysis provided several clues on regulation by specific transcription factors, with emerged with high Z-scores on much smaller numbers of putative targets. Noteworthy was the high Z-score of Pax5 and Pax6 for posterior genes (8 and 5 gene hits, respectively; *Crabp2*, *Dbx1*, *Rarb* and *Stra6* appearing as putative Pax6 targets). Regulation by Pbx1, a cofactor for Hox genes, was also highlighted for 15 posterior genes (Table 3). These included already documented Hox/Pbx targets such as *Hoxa1* [80] and *Rarb* [81], and pointed to *Ccne1*, *Lrg5*, *Nepn*, *NKx2-9* and *3-1* or *Rfx6* as additional putative targets. A set of 8 genes was defined as putative Brachyury (T) targets; these included *Lhx1*, *Pmm2*, *Prdm13*, *NKx2-9* and *Stra6*. Involvement of STAT1 and/or STAT3 as possible regulators of several posterior genes was highlighted. These included *Ccne1*, *Crabp2*, *Dbx1*, *Dhrs3*, *Rarb* and *Stra6*. Finally, three nuclear receptors were identified through this analysis: NR2F1 (COUP-TF I) as a putative regulator of 17 posterior genes, the PPARγ:RXRα dimer, with 22 putative targets among posterior genes, and NR3C1 (glucocorticoid receptor) with 21 identified targets among anterior genes (details on target genes in Table 3).

Altogether, this TFBS database search highlighted the combinatorial regulation of embryonic RA-dependent genes by several families of transcriptional regulators, while providing strong candidates for the regulation of specific subsets of genes. It also pointed out to four genes of the RA pathway: *Dhrs3*, *Crabp2*, *Rarb*, and *Stra6*, as key targets for such combinatorial regulation.

### Conclusion

This study provides an overview of the genes misregulated in early mouse embryos unable to synthesize retinoic acid (RA) from its precursor retinaldehyde, due to targeted gene disruption of a critical biosynthesis enzyme. While highlighting several genes known to be affected by conditions of impaired RA signaling in mouse embryos and/or other animal models, it provides additional candidate genes and pathways that may be relevant to better understand the mechanisms underlying the complex phenotypic consequences of embryonic RA deficiency. Bioinformatic analysis confirmed the impact of retinoid deficiency on several signaling pathways, with specific genes misregulated and belonging to the FGF, Notch, BMP, Hedgehog, VEGF, or Wnt pathways. This analysis pointed out to additional pathways or biological functions which, although not previously correlated with retinoid deficiency, appeared in the top (most significant) categories. This provides clues for further exploration of the effect of retinoid deficiency on metabolic processes such as purine and pyrimidine metabolism or one carbon metabolism, on signaling pathways such as the mTOR, ILK and IGF1 pathways, or on protein ubiquitination. Our data also suggest an upregulation of calcium signaling, specific to posterior (trunk) tissues of RA-deficient embryos.

This study adds to a framework of other high-throughput studies that reported RA-dependent transcriptomic alterations [72], [73], [74], [82], or characterization of RAR target gene loci [72], [75], [76], [82], in tissue culture cells including embryonic stem cells. Remarkably, a cross comparison between several data sets revealed that only a small subset of genes downregulated in *Raldh2* ^−/−^ embryos were previously identified as RAR-bound, or RA-inducible, in embryonic stem cells. Future high-throughput genomic/transcriptomic studies, expected to be performed on other retinoid target tissues, should improve our knowledge on retinoid-dependent gene regulation, both during normal developmental and physiological processes, and with respect to various diseases including cancer.

## Materials and Methods

### Ethics statement, mouse lines and embryonic tissue collection

Animal experimentation protocols were reviewed and approved by the Direction Départementale des Services Vétérinaires (agreement # 67–189 dated March 30, 2009) and conformed to the NIH and European Union guidelines, provisions of the Guide for the Care and Use of Laboratory Animals, and the Animal Welfare Act. No invasive (pain-inducing) procedure was used for this study. The mice harbored the *Raldh2* knockout allele described in ref. [26] and, for the X-gal assays, the RARE-hsp68-*lacZ* transgene described in ref. [32]. Pregnant female mice were euthanized after anesthesia with CO_2_, embryos were collected and tissue samples were quickly dissected, by sectioning with fine tweezers and freezing in liquid nitrogen. Extra-embryonic membranes were used for genotyping of the embryos.

### Microarray hybridization and bioinformatic analysis

Total RNA was extracted with the RNAeasy micro Kit (Qiagen) from individual embryonic samples. RNA quality was verified by analysis on a 2100 Bioanalyzer (Agilent), and only samples displaying a RNA Integrity Number (RIN) greater than 9.0 were selected for analysis. Biotinylated single strand cDNA targets were prepared, starting from 300 ng RNA, using the Ambion WT Expression Kit (Cat #4411974) and the Affymetrix GeneChip WT Terminal Labeling Kit (Cat #900671), according to Affymetrix recommendations. For each sample, 1.9 μg cDNA was hybridized for 16 h at 45°C on GeneChip Mouse Gene 1.0 ST arrays, containing 35556 probe sets interrogating over 28000 genes represented by 27 probes (25-mer) spread across the full length of the gene. The chips were washed and stained in the GeneChip Fluidics Station 450 (Affymetrix), and scanned with the GeneChip Scanner 3000 7G (Affymetrix). Finally, raw data (.CEL Intensity files) were extracted from the scanned images using the Affymetrix GeneChip Command Console (AGCC) version 3.1.

CEL files were further processed with the Partek genomics suite 6.5 software to obtain principal component analysis (PCA), in order to verify the sample distribution. Genes considered as differentially expressed had a hybridization signal value above 5 (20^th^ percentile of all expression values) in at least one sample. Differential expression was considered significant if the false discovery rate from Benjamini and Hochberg test was under 10%, corresponding to a p-value ≤0.05 for the ANT data set and ≤0.03 for the POST data set, determined by student t-test. Selected lists for the ANT and the POST data sets were analyzed with the Ingenuity pathway analysis (http://www.Ingenuity.com) software. The array CEL files were submitted to the NIH Gene Expression Omnibus (GEO) database (accession number GEO: GSE43578).

### Real-time quantitative RT-PCR

RT-PCR assays were performed in triplicate on three independent RNA samples for each tissue type and genotype. RNA extractions were performed as previously described. Oligo-dT primed cDNAs were generated using the Superscript II kit (Invitrogen) according to the manufacturer's protocol with. Quantitative real-time PCR was achieved using SybrGreen and LightCycler 480 (Roche). The sequences of primers used for the various tested genes are given in Table S4 (online supporting information). Probe sets for detection of mouse *Gapdh* (glyceraldehyde-3-phosphate dehydrogenase) and *Actb* (beta-actin) were used for normalization. For each sample the ratio between signals for the gene of interest and *Gapdh* was calculated to normalize concentration values. To verify if genes were differentially expressed, the average of ratios calculated for wild-type and mutant samples were then compared.

### In situ hybridization and X-gal staining

Whole-mount in situ hybridization with digoxigenin-labeled riboprobes was performed as described [83], using an Intavis InSituPro robot (for details, see http://www.empress.har.mrc.ac.uk/browser/, Gene Expression section). X-gal assays were performed as described [32].

## Supporting Information

Figure S1 **Hierarchical clustering of differentially expressed genes.** The heatmap shows the gene expression profiles of microarray data sets (upregulated expression: red; downregulated expression: green). Importantly, the WT and KO samples segregate into fully distinct clusters according to this analysis. ANT: RNA from anterior tissues; POST: RNA from posterior tissues; WT: wild-type embryos; KO: *Raldh2* ^−/−^ embryos.
(TIF)

Table S1 **Genes exhibiting a “contradictory” behavior in the microarray experiments, i.e. downregulated in one type of tissue samples and upregulated in the other (±1.2 fold change) in** ***Raldh2*** **^−/−^ embryos.**
(DOC)

Table S2 **Genes involved in the process of transcription/transcriptional regulation and downregulated in** ***Raldh2*** **^−/−^ embryos.** Genes are listed separately for the ANT and POST microarray experiments. Genes with <−1.5 fold changes appear in bold.
(PDF)

Table S3 **Genes found in common in our transcriptomic study (genes downregulated in anterior or posterior tissues of** ***Raldh2*** **^−/−^ embryos), and in studies which identified RA-inducible genes by RNA-seq (ref.** [**72**] **) or microarray (refs.** [**73**], [**74**] **) analysis of differentiating ES cells.** A cross-comparison with genes reported as RAR-bound in differentiating ES cells (ref. [72]) was also performed.
(PDF)

Table S4 **Sequences of primers used in the quantitative RT-PCR experiments (F: forward primer; R: reverse primer).**
(PDF)
