# Supplementary figures and images for: Transcriptomic Analysis of Murine Embryos Lacking Endogenous Retinoic Acid Signaling
Source: PLoS One. 2013 Apr 24;8(4):e62274. doi: 10.1371/journal.pone.0062274 (PMC3634737; doi:10.1371/journal.pone.0062274)

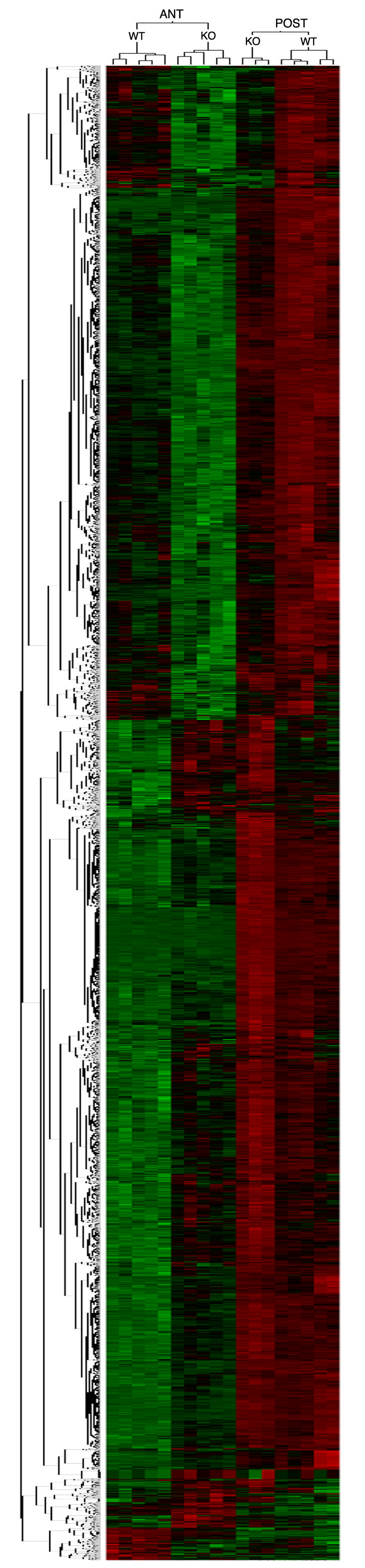

Supplement: Figure S1 — Hierarchical clustering of differentially expressed genes. The heatmap shows the gene expression profiles of microarray data sets (upregulated expression: red; downregulated expression: green). Importantly, the WT and KO samples segregate into fully distinct clusters according to this analysis. ANT: RNA from anterior tissues; POST: RNA from posterior tissues; WT: wild-type embryos; KO: Raldh2 −/− embryos. (TIF) [file pone.0062274.s001.tif]
