# Supplementary material for: Transcriptomic Analysis of Murine Embryos Lacking Endogenous Retinoic Acid Signaling
Source: PLoS One. 2013 Apr 24;8(4):e62274. doi: 10.1371/journal.pone.0062274 (PMC3634737; doi:10.1371/journal.pone.0062274)
Supplement: Table S1 — Genes exhibiting a “contradictory” behavior in the microarray experiments, i.e. downregulated in one type of tissue samples and upregulated in the other (±1.2 fold change) in Raldh2 −/− embryos. (DOC) [file pone.0062274.s002.doc]

**Table S1**

|  |  | **Symbol** | **Name** |  |
| --- | --- | --- | --- | --- |
|  |  |  |  |  |
| **DOWN in** |  | *Cacna2d2* | Calcium channel a2/d2 subunit |  |
| **ANTERIOR** |  | *Cap2* | CAP, adenylate cyclase-associated protein |  |
| **/ UP in** |  | *Col14a1* | Collagen, type 14 |  |
| **POSTERIOR** |  | *Epha4* | Eph receptor A4 |  |
| **TISSUES** |  | *Lphn2* | Latrophilin 2 |  |
|  |  | *Lrrn4* | Leucine rich repeat neuronal 4 |  |
|  |  | *Mapk10* | Mitogen-activated protein kinase 10 |  |
|  |  | *Me1* | Malic enzyme 1 |  |
|  |  | *Mecom* | MDS1 and EVI1 complex locus |  |
|  |  | *Msrb3* | Methionine sulfoxide reductase B3 |  |
|  |  | *Plagl1* | Pleiomorphic adenoma gene-like 1 |  |
|  |  | *Rprm* | Reprimo, TP53 dependent G2 arrest mediator candidate |  |
|  |  |  |  |  |
|  |  |  |  |  |
| **UP in** |  | *Cldn4* | Claudin 4 |  |
| **ANTERIOR** |  | *Dhrs3* | Dehydrogenase/reductase (SDR family) 3 |  |
| **/ DOWN in** |  | *Gprc5a* | G protein-coupled receptor C5A |  |
| **POSTERIOR** |  | *Ifrd1* | Interferon-related developmental regulator 1 |  |
| **TISSUES** |  | *Lgr5* | Leucine rich repeat containing G protein coupled receptor 5 |  |
|  |  | *Lrrtm3* | Leucine rich repeat transmembrane neuronal 3 |  |
|  |  | *Mapk13* | Mitogen-activated protein kinase 13 |  |
|  |  | *Meox1* | Mesenchyme homeobox 1 |  |
|  |  | *Mtap7* | Microtubule-associated protein 7 domain containing 1 |  |
|  |  | *Parvb* | Parvin, beta |  |
|  |  | *Phlda1* | Pleckstrin homology-like domain A1 |  |
|  |  | *Prdm1* | PR domain containing 1, with ZNF domain |  |
|  |  | *Ptges* | Prostaglandin E synthase |  |
|  |  | *Stk32a* | Serine/threonine kinase 32A |  |
|  |  | *Vsnl1* | Visinin-like 1 |  |
|  |  |  |  |  |
