# Supplementary material for: Transcriptomic Analysis of Murine Embryos Lacking Endogenous Retinoic Acid Signaling
Source: PLoS One. 2013 Apr 24;8(4):e62274. doi: 10.1371/journal.pone.0062274 (PMC3634737; doi:10.1371/journal.pone.0062274)
Supplement: Table S2 — Genes involved in the process of transcription/transcriptional regulation and downregulated in Raldh2 −/− embryos. Genes are listed separately for the ANT and POST microarray experiments. Genes with <−1.5 fold changes appear in bold. (PDF) [file pone.0062274.s003.pdf]

Downregulated in ANTERIOR tissues

Abcg4	ATP-binding cassette, sub-family G, member 4
Arntl	aryl hydrocarbon receptor nuclear translocator-like
Arx	aristaless related homeobox
Asf1a	ASF1 anti-silencing function 1 homolog A (S. cerevisiae)
Asf1b	ASF1 anti-silencing function 1 homolog B (S. cerevisiae)
Atf6b	activating transcription factor 6 beta
Atf7ip2	activating transcription factor 7 interacting protein 2
AW146020	expressed sequence AW146020
Baz1a	bromodomain adjacent to zinc finger domain 1A
Bclaf1	BCL2-associated transcription factor 1
Bmi1	Bmi1 polycomb ring finger oncogene
Bzw1	basic leucine zipper and W2 domains 1
Ccar1	cell division cycle and apoptosis regulator 1
Ccdc101	coiled-coil domain containing 101
Ccnc	cyclin C
Chmp1a	chromatin modifying protein 1A
Cnot6	CCR4-NOT transcription complex, subunit 6
Cops5	COP9 homolog, subunit 5 (Arabidopsis thaliana)
Crcp	calcitonin gene-related peptide-receptor component protein
Ctcf	CCCTC-binding factor
Cxxc1	CXXC finger 1 (PHD domain)
Cytl1	**cytokine-like 1**
Dach1	dachshund 1 (Drosophila)
Ddx20	DEAD (Asp-Glu-Ala-Asp) box polypeptide 20
Dido1	death inducer-obliterator 1
Dpf1	D4, zinc and double PHD fingers family 1
E2f2	E2F transcription factor 2
E2f3	E2F transcription factor 3
E2f4	E2F transcription factor 4
E2f8	E2F transcription factor 8
Ecd	ecdysoneless homolog (Drosophila)
Ecsit	ECSIT homolog (Drosophila)
Eid3	**EP300 interacting inhibitor of differentiation 3**
Elof1	elongation factor 1 homolog (ELF1, S. cerevisiae)
Emx2	**empty spiracles homolog 2 (Drosophila)**
Eny2	enhancer of yellow 2 homolog (Drosophila)
Ercc8	excision repair cross-complementing repair deficiency, group 8
Etv4	ets variant gene 4 (E1A enhancer binding protein, E1AF)
EU599041	expressed sequence EU599041
Fezf2	Fez family zinc finger 2
Foxg1	forkhead box G1
Gabpb2	GA repeat binding protein, beta 2
Gfi1b	growth factor independent 1B
Gm1008	predicted gene 1008
Gtf2f1	general transcription factor IIF, polypeptide 1
Gtf2f2	general transcription factor IIF, polypeptide 2
Gtf2h5	general transcription factor IIH, polypeptide 5
Gtf3c6	**general transcription factor IIIC, polypeptide 6, alpha**
Hdx	highly divergent homeobox
Hells	helicase, lymphoid specific
Hes5	hairy and enhancer of split 5 (Drosophila)
Hexim1	hexamethylene bis-acetamide inducible 1
Hira	histone cell cycle regulation defective homolog A (S. cerevisiae)
Hnrpdl	heterogeneous nuclear ribonucleoprotein D-like
Ikzf1	**IKAROS family zinc finger 1**
Irak1bp1	interleukin-1 receptor-associated kinase 1 binding protein 1
Kcnh2	potassium voltage-gated channel, subfamily H (eag-related), member 2
Klf1	Kruppel-like factor 1 (erythroid)
L3mbtl2	l(3)mbt-like 2 (Drosophila)
Lhx2	**LIM homeobox protein 2**
Mapk8ip1	mitogen-activated protein kinase 8 interacting protein 1
Meaf6	MYST/Esa1-associated factor 6
Med10	mediator of RNA polymerase II transcription, subunit 10 homolog (yeast)
Med18	mediator of RNA polymerase II transcription, subunit 18 homolog (yeast)
Med27	mediator complex subunit 27
Med4	mediator of RNA polymerase II transcription, subunit 4 homolog (yeast)
Med8	mediator of RNA polymerase II transcription, subunit 8 homolog (yeast)
Mlf1ip	myeloid leukemia factor 1 interacting protein
Msl3	male-specific lethal 3 homolog (Drosophila)
Myb	myeloblastosis oncogene
Narg1	NMDA receptor-regulated gene 1
Ncoa5	nuclear receptor coactivator 5
Nfatc2ip	nuclear factor of activated T-cells, calcineurin-dependent 2 interacting
Nfyb	nuclear transcription factor-Y beta
Nkrf	**NF-kappaB repressing factor**
Npm3	nucleoplasmin 3
Nr2e1	**nuclear receptor subfamily 2, group E, member 1**
Pa2g4	proliferation-associated 2G4
Phf5a	PHD finger protein 5A
Pkia	**protein kinase inhibitor, alpha**
Pkig	protein kinase inhibitor, gamma
Plagl1	pleiomorphic adenoma gene-like 1
Pnn	pinin
Polr1b	polymerase (RNA) I polypeptide B
Polr1e	**polymerase (RNA) I polypeptide E**
Polr2d	polymerase (RNA) II (DNA directed) polypeptide D
Polr2h	polymerase (RNA) II (DNA directed) polypeptide H
Polr2l	polymerase (RNA) II (DNA directed) polypeptide L
Polr2l	polymerase (RNA) II (DNA directed) polypeptide L
Polr3e	polymerase (RNA) III (DNA directed) polypeptide E
Pou3f3	**POU domain, class 3, transcription factor 3**
Ppp1r10	protein phosphatase 1, regulatory subunit 10
Preb	prolactin regulatory element binding
Prim1	DNA primase, p49 subunit
Prmt6	protein arginine N-methyltransferase 6
Psmc5	protease (prosome, macropain) 26S subunit, ATPase 5
Ptges2	prostaglandin E synthase 2
Purb	purine rich element binding protein B
Pus1	pseudouridine synthase 1
Rrn3	RRN3 RNA polymerase I transcription factor homolog (yeast)
Sap18	Sin3-associated polypeptide 18
Sap30bp	SAP30 binding protein
Satb2	special AT-rich sequence binding protein 2
Setd1b	SET domain containing 1B
Sfmbt1	Scm-like with four mbt domains 1

Downregulated in POSTERIOR tissues

Asf1b	ASF1 anti-silencing function 1 homolog B (S. cerevisiae)
Baz1a	bromodomain adjacent to zinc finger domain 1A
Ccdc101	coiled-coil domain containing 101
Ccnc	cyclin C
Cdx1	**caudal type homeo box 1**
Crcp	calcitonin gene-related peptide-receptor component protein
Cxxc1	CXXC finger 1 (PHD domain)
Dbx1	developing brain homeobox 1
Dr1	down-regulator of transcription 1
E2f3	E2F transcription factor 3
E2f4	E2F transcription factor 4
E2f8	E2F transcription factor 8
Ecsit	ECSIT homolog (Drosophila)
Edf1	endothelial differentiation-related factor 1
Elof1	elongation factor 1 homolog (ELF1, S. cerevisiae)
Ercc8	excision repaiross-complementing rodent repair deficiency, group 8
Foxa1	forkhead box A1
Foxd3	forkhead box D3
Foxp4	forkhead box P4
Gli1	GLI-Kruppel family member GLI1
Gtf2f1	general transcription factor IIF, polypeptide 1
Gtf2f2	general transcription factor IIF, polypeptide 2
Gtf3c6	general transcription factor IIIC, polypeptide 6, alpha
Hoxa1	**homeo box A1**
Hoxb1	homeo box B1
Irak1bp1	**interleukin-1 receptor-associated kinase 1 binding protein 1**
L3mbtl2	l(3)mbt-like 2 (Drosophila)
Lhx1	**LIM homeobox protein 1**
Med22	mediator complex subunit 22
Med27	mediator complex subunit 27
Med29	mediator complex subunit 29
Med8	mediator of RNA polymerase II transcription, subunit 8 homolog (yeast)
Meox1	**mesenchyme homeobox 1**
Mlf1ip	myeloid leukemia factor 1 interacting protein
Mlx	MAX-like protein X
Myb	myeloblastosis oncogene
Narg1	NMDA receptor-regulated gene 1
Nepn	**nephrocan**
Nfatc2ip	nuclear factor of activated T-cells, calcineurin-dependent 2 interacting
Nkrf	NF-kappaB repressing factor
Nkx2-9	**NK2 transcription factor related, locus 9 (Drosophila)**
Nkx3-1	**NK-3 transcription factor, locus 1 (Drosophila)**
Npm3	nucleoplasmin 3
Pa2g4	proliferation-associated 2G4
Pax6	**paired box gene 6**
Pcgf6	polycomb group ring finger 6
Pdx1	pancreatic and duodenal homeobox 1
Polr1b	polymerase (RNA) I polypeptide B
Polr1d	polymerase (RNA) I polypeptide D
Polr1e	polymerase (RNA) I polypeptide E
Polr2h	polymerase (RNA) II (DNA directed) polypeptide H
Polr2i	polymerase (RNA) II (DNA directed) polypeptide I
Polr3d	polymerase (RNA) III (DNA directed) polypeptide D
Pprc1	peroxisome proliferative activated receptor gamma, coactivator-related 1
Prdm1	PR domain containing 1, with ZNF domain
Preb	prolactin regulatory element binding
Psmc5	protease (prosome, macropain) 26S subunit, ATPase 5
Ptges2	prostaglandin E synthase 2
Pus1	pseudouridine synthase 1
Rarb	**retinoic acid receptor, beta**
Rel	reticuloendotheliosis oncogene
Rfx6	**regulatory factor X, 6**
Rrn3	RRN3 RNA polymerase I transcription factor homolog (yeast)
Sall2	sal-like 2 (Drosophila)
Sfmbt1	Scm-like with four mbt domains 1
Sim1	single-minded homolog 1 (Drosophila)
Snf8	SNF8, ESCRT-II complex subunit, homolog (S. cerevisiae)
Sox3	SRY-box containing gene 3
Taf12	TAF12, TATA box binding protein (TBP)-associated factor
Taf13	TAF13, TATA box binding protein (TBP)-associated factor
Taf1d	TATA box binding protein (Tbp)-associated factor, RNA polymerase I, D
Taf4b	TAF4B, TATA box binding protein (TBP)-associated factor
Taf9	TAF9, TATA box binding protein (TBP)-associated factor
Taf9b	TAF9B, TATA box binding protein (TBP)-associated factor
Tcf15	transcription factor 15
Tcof1	Treacher Collins Franceschetti syndrome 1, homolog
Tfb2m	transcription factor B2, mitochondrial
Topors	topoisomerase I binding, arginine/serine-rich
Uimc1	ubiquitin interaction motif containing 1
Usp16	ubiquitin specific peptidase 16
Vps36	vacuolar protein sorting 36 (yeast)
Zc3h8	zinc finger CCCH type containing 8

Shox2	**short stature homeobox 2**
Six2	**sine oculis-related homeobox 2 homolog (Drosophila)**
Smarcd3	SWI/SNF related, matrix associated, actin dependent regulator of chromatin, subfamily d, member 3
Snf8	SNF8, ESCRT-II complex subunit, homolog (S. cerevisiae)
Sox1	SRY-box containing gene 1
Sox21	SRY-box containing gene 21
Sox3	SRY-box containing gene 3
Stat1	signal transducer and activator of transcription 1
Stat4	**signal transducer and activator of transcription 4**
Tada2l	transcriptional adaptor 2 (ADA2 homolog, yeast)-like
Taf1	TAF1, TATA box binding protein (TBP)-associated factor
Taf11	TAF11, TATA box binding protein (TBP)-associated factor
Taf12	TAF12, TATA box binding protein (TBP)-associated factor
Taf13	TAF13, TATA box binding protein (TBP)-associated factor
Taf1d	**TATA box binding protein (Tbp)-associated factor, RNA polymerase I, D**
Taf9b	**TAF9B RNA polymerase II, TATA box binding protein (TBP)-associated factor**
Tardbp	TAR DNA binding protein
Tbpl1	TATA box binding protein-like 1
Tcfec	transcription factor EC
Tfb2m	transcription factor B2, mitochondrial
Traf3ip1	TRAF3 interacting protein 1
Traf7	TNF receptor-associated factor 7
Ttll5	tubulin tyrosine ligase-like family, member 5
Ubtf	upstream binding transcription factor, RNA polymerase I
Usp16	ubiquitin specific peptidase 16
Vps36	vacuolar protein sorting 36 (yeast)
Wdr77	WD repeat domain 77
Yeats4	YEATS domain containing 4
Zfp251	zinc finger protein 251
Zfp322a	zinc finger protein 322A
Zfp326	zinc finger protein 326
Zfp422	zinc finger protein 422
Zfp553	zinc finger protein 553
Zfp689	zinc finger protein 689
Zhx3	zinc fingers and homeoboxes 3
Zic3	zinc finger protein of the cerebellum 3
Znfx1	zinc finger, NFX1-type containing 1
Zscan22	zinc finger and SCAN domain containing 22
