# Supplementary material for: Transcriptomic Analysis of Murine Embryos Lacking Endogenous Retinoic Acid Signaling
Source: PLoS One. 2013 Apr 24;8(4):e62274. doi: 10.1371/journal.pone.0062274 (PMC3634737; doi:10.1371/journal.pone.0062274)
Supplement: Table S3 — Genes found in common in our transcriptomic study (genes downregulated in anterior or posterior tissues of Raldh2 −/− embryos), and in studies which identified RA-inducible genes by RNA-seq (ref. [72] ) or microarray (refs. [73], [74] ) analysis of differentiating ES cells. A cross-comparison with genes reported as RAR-bound in differentiating ES cells (ref. [72]) was also performed. (PDF) [file pone.0062274.s004.pdf]

Cross-comparison of genes downregulated in ANTERIOR tissues

RAR-bound loci in ES cells (ChIP-seq: ref. 72) 31 genes

Acsl4	acyl-CoA synthetase long-chain family member 4
Ak2	similar to adenylate kinase 2; adenylate kinase 2
Ankrd9	ankyrin repeat domain 9
Apex1	apurinic/apyrimidinic endonuclease 1
Atp1b1	ATPase, Na+/K+ transporting, beta 1 polypeptide
Bmpr1b	bone morphogenetic protein receptor, type 1B
Brp44l	similar to brain protein 44-like protein
Camk2a	calcium/calmodulin-dependent protein kinase II alpha
Ccar1	cell division cycle and apoptosis regulator 1
Cfl2	cofilin 2, muscle
Cpt1a	carnitine palmitoyltransferase 1a, liver
E2f2	E2F transcription factor 2
Fabp5	fatty acid binding protein 5, epidermal
Fam105b	family with sequence similarity 105, member B
Gca	grancalcin
Glt1d1	glycosyltransferase 1 domain containing 1
Gnb1l	guanine nucleotide binding protein (G protein)
Ly6c1	lymphocyte antigen 6 complex, locus C2
Mobkl1a	MOB1, Mps One Binder kinase activator-like 1A (yeast)
Mtap1a	microtubule-associated protein 1 A
Myg1	melanocyte proliferating gene 1
Ogdh	oxoglutarate dehydrogenase (lipoamide)
Ppih	similar to peptidyl prolyl isomerase H
Pptc7	PTC7 protein phosphatase homolog (S. cerevisiae)
Rbm34	RNA binding motif protein 34
Rnf144b	ring finger protein 144B
Slc35f2	solute carrier family 35, member F2
Znfx1	zinc finger, NFX1-type containing 1
1300001I01Rik	RIKEN cDNA 1300001I01 gene
2010305A19Rik	RIKEN cDNA 2010305A19 gene
9630033F20Rik	RIKEN cDNA 9630033F20 gene

RA-induced in ES cells (RNA-seq: ref. 72) 56 genes

Acsl4	acyl-CoA synthetase long-chain family member 4
Apc2	adenomatosis polyposis coli 2
Arsb	arylsulfatase B
Atp1b1	ATPase, Na+/K+ transporting, beta 1 polypeptide
Baz1a	bromodomain adjacent to zinc finger domain 1A
Car3	carbonic anhydrase 3
Clgn	calmegin
Crabp2	cellular retinoic acid binding protein II
Csn3	casein kappa
Cyp4f15	cytochrome P450, family 4, subfamily f, polypeptide 15
Dach1	dachshund 1 (Drosophila)
Dleu7	deleted in lymphocytic leukemia, 7
Dll1	delta-like 1 (Drosophila)
Dpysl4	dihydropyrimidinase-like 4
Elovl2	elongation of very long chain fatty acids (Elo2/Elo3, yeast)-like 2
Eml1	echinoderm microtubule associated protein like 1
Enpp4	ectonucleotide pyrophosphatase/phosphodiesterase 4
Epha4	Eph receptor A4
Fam20c	family with sequence similarity 20, member C
Fez1	fasciculation and elongation protein zeta 1 (zygin I)
Fhod3	formin homology 2 domain containing 3
Fktn	fukutin
Foxg1	forkhead box G1
Hes5	hairy and enhancer of split 5 (Drosophila)
Ildr2	immunoglobulin-like domain containing receptor 2
Lhx2	LIM homeobox protein 2
Lrig1	leucine-rich repeats and immunoglobulin-like domains 1
Mecom	MDS1 and EVI1 complex locus
Metrn	meteorin, glial cell differentiation regulator
Mpped1	metallophosphoesterase domain containing 1
Mras	muscle and microspikes RAS
Msrb3	methionine sulfoxide reductase B3
Mtmr1	myotubularin related protein 1
Nat8l	N-acetyltransferase 8-like
Pcsk9	proprotein convertase subtilisin/kexin type 9
Pklr	pyruvate kinase liver and red blood cell
Plagl1	pleiomorphic adenoma gene-like 1
Ppm1e	protein phosphatase 1E (PP2C domain containing)
Ppp2r2c	protein phosphatase 2, regulatory subunit B, gamma isoform
Ptn	pleiotrophin
Ptprz1	protein tyrosine phosphatase, receptor type Z, polypeptide 1
Rbks	ribokinase
Rprm	reprimo, TP53 dependent G2 arrest mediator candidate
Slc35f1	solute carrier family 35, member F1
Slc46a1	solute carrier family 46, member 1
Slco3a1	solute carrier organic anion transporter family, member 3a1
Smox	spermine oxidase
Sox21	SRY-box containing gene 21
Spef1	sperm flagellar 1
Ssh2	slingshot homolog 2 (Drosophila)
Tcf19	transcription factor 19
Unc5a	unc-5 homolog A (C. elegans)
Vash1	vasohibin 1
Wnt7b	wingless-related MMTV integration site 7B
3010003L21Rik	RIKEN cDNA 3010003L21 gene
6330407J23Rik	RIKEN cDNA 6330407J23 gene

Cross-comparison of genes downregulated in POSTERIOR tissues

RAR-bound loci in ES cells (ChIP-seq: ref. 72) 15 genes

Dhrs3	dehydrogenase/reductase (SDR family) member 3
Fam105b	family with sequence similarity 105, member B
Gar1	GAR1 ribonucleoprotein homolog (yeast)
Gnb1l	guanine nucleotide binding protein, beta polypeptide 1-like
Pptc7	PTC7 protein phosphatase homolog (S. cerevisiae)
Ptcd3	pentatricopeptide repeat domain 3
Uck1	uridine-cytidine kinase 1; predicted gene 4482
Zfp428	zinc finger protein 428
BC048403	cDNA sequence BC048403
D19Bwg1357e	DNA segment, Chr 19, Brigham & Women's Genetics 1357
E130309F12Rik	RIKEN cDNA E130309F12 gene
1300001I01Rik	RIKEN cDNA 1300001I01 gene
2010305A19Rik	RIKEN cDNA 2010305A19 gene
4631416L12Rik	RIKEN cDNA 4631416L12 gene
9630033F20Rik	RIKEN cDNA 9630033F20 gene

RA-induced in ES cells (RNA-seq: ref. 72) 48 genes

Alpl	alkaline phosphatase, liver/bone/kidney
Arg1	arginase, liver
Baz1a	bromodomain adjacent to zinc finger domain 1A
Casp8	caspase 8
Cdx1	caudal type homeobox 1
Cpn1	carboxypeptidase N, polypeptide 1
Crabp2	cellular retinoic acid binding protein II
Csn3	casein kappa
Dbx1	developing brain homeobox 1
Ddc	dopa decarboxylase
Dhrs3	dehydrogenase/reductase (SDR family) member 3
Dleu7	deleted in lymphocytic leukemia, 7
Dusp5	dual specificity phosphatase 5
Elovl2	elongation of very long chain fatty acids (Elo2/Elo3, yeast)-like 2
Fndc5	fibronectin type III domain containing 5
Foxa1	forkhead box A1
Fzd4	frizzled homolog 4 (Drosophila)
Gprc5a	G protein-coupled receptor, family C, group 5, member A
Hoxa1	homeobox A1
Hoxb1	homeobox B1
Kcng1	potassium voltage-gated channel, subfamily G, member 1
Kynu	kynureninase (L-kynurenine hydrolase)
Lhx1	LIM homeobox protein 1
Lrig1	leucine-rich repeats and immunoglobulin-like domains 1
Maob	monoamine oxidase B
Metrn	meteorin, glial cell differentiation regulator
Mtmr1	myotubularin related protein 1
Nat8l	N-acetyltransferase 8-like
Nkx3-1	NK-3 transcription factor, locus 1 (Drosophila)
Nrcam	neuron-glia-CAM-related cell adhesion molecule
Nudt10	nudix (nucleoside diphosphate linked moiety X)-type motif 10
Pax6	paired box gene 6
Prdm1	PR domain containing 1, with ZNF domain
Ptges	prostaglandin E synthase
Ptprz1	protein tyrosine phosphatase, receptor type Z, polypeptide 1
Rarb	retinoic acid receptor, beta
Sfrp5	secreted frizzled-related sequence protein 5
Slc35c1	solute carrier family 35, member C1
Smox	spermine oxidase
Stk32a	serine/threonine kinase 32A
Stra6	stimulated by retinoic acid gene 6
Tmem200b	transmembrane protein 200B
Tnfrsf21	tumor necrosis factor receptor superfamily, member 21
Uck1	uridine-cytidine kinase 1
Vash1	vasohibin 1
E130309F12Rik	RIKEN cDNA E130309F12 gene
E130012A19Rik	RIKEN cDNA E130012A19 gene
1700011H14Rik	RIKEN cDNA 1700011H14 gene

Induced in differentiating ES cells (arrays: ref. 73)   75 genes

Ankrd13c	ankyrin repeat domain 13c
Armc6	armadillo repeat containing 6
Asb13	ankyrin repeat and SOCS box-containing 13
Cacna2d2	calcium channel, voltage-dependent, alpha 2/delta subunit 2
Capn7	calpain 7
Ccdc101	coiled-coil domain containing 101
Cdh7	cadherin 7, type 2
Cend1	cell cycle exit and neuronal differentiation 1
Cltb	clathrin, light polypeptide (Lcb)
Cops5	COP9 (constitutive photomorphogenic) homolog, subunit 5
Crcp	calcitonin gene-related peptide-receptor component protein
Cycs	cytochrome c, somatic
Cyp46a1	cytochrome P450, family 46, subfamily a, polypeptide 1
D16H22S680E	DNA segment, Chr 16, human D22S680E, expressed
Dock3	dedicator of cyto-kinesis 3
Ehd3	EH-domain containing 3
Ercc8	excision repair cross-complementing, complementation group 8
Freq	frequenin homolog (Drosophila)
Hras1	Harvey rat sarcoma virus oncogene 1
Hsd17b7	hydroxysteroid (17-beta) dehydrogenase 7
Idh3a	isocitrate dehydrogenase 3 (NAD+) alpha
Klhl18	kelch-like 18 (Drosophila)
Kpna4	karyopherin (importin) alpha 4
Limk1	LIM-domain containing, protein kinase
Lmo2	LIM domain only 2
Lonrf2	LON peptidase N-terminal domain and ring finger 2
Lrrc4	leucine rich repeat containing 4
Man1a2	mannosidase, alpha, class 1A, member 2
Me1	malic enzyme 1, NADP(+)-dependent, cytosolic
Mfn2	mitofusin 2
Mvk	mevalonate kinase
Ndufa5	NADH dehydrogenase (ubiquinone) 1 alpha subcomplex, 5
Nkrf	NF-kappaB repressing factor
Nt5dc3	5'-nucleotidase domain containing 3
Nudt19	nudix (nucleoside diphosphate linked moiety X)-type motif 19
Olfm3	olfactomedin 3
Opn3	opsin 3
Pank1	pantothenate kinase 1
Pdxk	pyridoxal (pyridoxine, vitamin B6) kinase
Pitpnm1	phosphatidylinositol transfer protein, membrane-associated 1
Plaa	phospholipase A2, activating protein
Plxnc1	plexin C1
Pmvk	phosphomevalonate kinase
Polr2d	polymerase (RNA) II (DNA directed) polypeptide D
Pon2	paraoxonase 2
Ppp1r14b	protein phosphatase 1, regulatory (inhibitor) subunit 14B
Prdx3	peroxiredoxin 3
Prss23	protease, serine, 23
Psmd6	proteasome (macropain) 26S subunit, non-ATPase, 6
Rab30	RAB30, member RAS oncogene family
Sap18	Sin3-associated polypeptide 18
Sec23ip	Sec23 interacting protein
Sfrs3	splicing factor, arginine/serine-rich 3 (SRp20)
Slc25a10	solute carrier family 25 (dicarboxylate transporter), member 10
Slmo1	slowmo homolog 1 (Drosophila)
Srp9	signal recognition particle 9
Stk16	serine/threonine kinase 16
Sucla2	succinate-Coenzyme A ligase, ADP-forming, beta subunit
Taf9b	TAF9B, TATA box binding protein (TBP)-associated factor
Tfb2m	transcription factor B2, mitochondrial
Timm9	translocase of inner mitochondrial membrane 9 homolog (yeast)
Tsr2	TSR2, 20S rRNA accumulation, homolog (S. cerevisiae)
Ube2l3	ubiquitin-conjugating enzyme E2L 3
Ublcp1	ubiquitin-like domain containing CTD phosphatase 1
Ugt8a	UDP galactosyltransferase 8A
Vps36	vacuolar protein sorting 36 (yeast)
Vwc2	von Willebrand factor C domain containing 2
Ykt6	YKT6 homolog (S. Cerevisiae)
Zcchc17	zinc finger, CCHC domain containing 17
Zfp326	zinc finger protein 326
1110049F12Rik	RIKEN cDNA 1110049F12 gene
2410004B18Rik	RIKEN cDNA 2410004B18 gene
2610028A01Rik	RIKEN cDNA 2610028A01 gene
4933433P14Rik	RIKEN cDNA 4933433P14 gene
6430704M03Rik	RIKEN cDNA 6430704M03 gene

Induced in differentiating ES cells (arrays: ref. 73)   42 genes

Armc6	armadillo repeat containing 6
Asb13	ankyrin repeat and SOCS box-containing 13
Bscl2	Bernardinelli-Seip congenital lipodystrophy 2 homolog (human)
Ccdc101	coiled-coil domain containing 101
Coq4	coenzyme Q4 homolog (yeast)
Crcp	calcitonin gene-related peptide-receptor component protein
Cycs	cytochrome c, somatic
D16H22S680E	DNA segment, Chr 16, human D22S680E, expressed
D17Wsu104e	DNA segment, Chr 17, Wayne State University 104, expressed
Dr1	down-regulator of transcription 1
Edf1	endothelial differentiation-related factor 1
Ercc8	excision repair cross-complementing, complementation group 8
Gpd1l	glycerol-3-phosphate dehydrogenase 1-like
Hras1	Harvey rat sarcoma virus oncogene 1
Lrrc4	leucine rich repeat containing 4
Lrrtm3	leucine rich repeat transmembrane neuronal 3
Mrps18a	mitochondrial ribosomal protein S18A
Mtap7d2	MAP7 domain containing 2
Nkrf	NF-kappaB repressing factor
Nt5dc3	5'-nucleotidase domain containing 3
Opn3	opsin 3
Pdxk	pyridoxal (pyridoxine, vitamin B6) kinase
Phlda1	pleckstrin homology-like domain, family A, member 1
Ppp1r14b	protein phosphatase 1, regulatory (inhibitor) subunit 14B
Psmd6	proteasome (macropain) 26S subunit, non-ATPase, 6
Sall2	sal-like 2 (Drosophila)
Slc25a10	solute carrier family 25 (dicarboxylate transporter), member 10
Slc25a33	solute carrier family 25, member 33
Taf9b	TAF9B, TATA box binding protein (TBP)-associated factor
Tfb2m	transcription factor B2, mitochondrial
Timm9	translocase of inner mitochondrial membrane 9 homolog (yeast)
Tsr2	TSR2, 20S rRNA accumulation, homolog (S. cerevisiae)
Usp46	ubiquitin specific peptidase 46
Vps36	vacuolar protein sorting 36 (yeast)
Ykt6	YKT6 homolog (S. Cerevisiae)
Zcchc17	zinc finger, CCHC domain containing 17
1110049F12Rik	RIKEN cDNA 1110049F12 gene
2300009A05Rik	RIKEN cDNA 2300009A05 gene
2400001E08Rik	RIKEN cDNA 2400001E08 gene
2410004B18Rik	RIKEN cDNA 2410004B18 gene
2610028A01Rik	RIKEN cDNA 2610028A01 gene
4933433P14Rik	RIKEN cDNA 4933433P14 gene

Induced in differentiating ES cells (arrays: ref. 74)   3 genes

Dido1	death inducer-obliterator 1
Farsa	phenylalanyl-tRNA synthetase, alpha subunit
Rpa2	replication protein A2

Induced in differentiating ES cells (arrays: ref. 74)   1 gene

Farsa	phenylalanyl-tRNA synthetase, alpha subunit
