# Supplementary material for: Transcriptomic Analysis of Murine Embryos Lacking Endogenous Retinoic Acid Signaling
Source: PLoS One. 2013 Apr 24;8(4):e62274. doi: 10.1371/journal.pone.0062274 (PMC3634737; doi:10.1371/journal.pone.0062274)
Supplement: Table S4 — Sequences of primers used in the quantitative RT-PCR experiments (F: forward primer; R: reverse primer). (PDF) [file pone.0062274.s005.pdf]

Gene	Sequence (5' →3')	Size (pb)
Pax6 F	ATGGGCGGAGTTATGATACCT	103
Pax6 R	GGTGAAATGAGTCCTGTTGAAGT	
Id4 F	CAGTGCGATATGAACGACTGC	72
Id4 R	GACTTTCTTGTTGGGCGGGAT	
Lhx2 F	CGCTCGGGACTTGGTTTATCA	112
Lhx2 R	CAAGCGGCAATAGACCAGG	
Six2 F	CTCACCACCACGCAAGTCAG	106
Six2 R	GGCTGCTGGAATTGGAGTTCT	
Stat4 F	GCAGCCAACATGCCTATCCA	95
Stat4 R	GCAGACACTTTGTGTTCCACAT	
Crabp2 F	ATGCCTAACTTTTCTGGCAACT	139
Crabp2 R	CCTGTTTGATCTCGACTGCTG	
Ikzf1 F	AGACAAGTGCCTGTCAGACAT	110
Ikzf1 R	CCAGGTAGTTGATGGCATTGTTG	
Unc5a F	CTCCTGGGCATAGTCCTCACT	156
Unc5a R	CAGCACGGGCTTGTTCTTG	
Bhlhe40 F	CTGTCAGGGATGGATTTTGCC	70
Bhlhe40 R	CTTCGCTCCGTTTTATTCCCC	
Creb5 F	GTCCCAGGCTCTCTATCATCTC	125
Creb5 R	ATAGGCATCAAGACGGCAGAA	
Actb F	AGTGTGACGTTGACATCCGTA	112
Actb R	GCCAGAGCAGTAATCTCCTTCT	
Gapdh F	AGGTCGGTGTGAACGGATTTG	123
Gapdh R	TGTAGACCATGTAGTTGAGGTCA	
